# ZIP10 drives osteosarcoma proliferation and chemoresistance through ITGA10-mediated activation of the PI3K/AKT pathway

**DOI:** 10.1186/s13046-021-02146-8

**Published:** 2021-10-27

**Authors:** Hongyu Li, Xin Shen, Mengjun Ma, Wenzhou Liu, Wen Yang, Peng Wang, Zhaopeng Cai, Rujia Mi, Yixuan Lu, Jiahao Zhuang, Yuhang Jiang, Yihui Song, Yanfeng Wu, Huiyong Shen

**Affiliations:** 1 Department of Orthopedics, The Eighth Affiliated Hospital, Sun Yat-sen University, No. 3025 Shennan Zhong Road, Shenzhen, Guangdong 518033 China; 2 Department of Orthopedics, Sun Yat-sen Memorial Hospital, Sun Yat-sen University, Guangzhou, 510000 China; 3 Center for Biotherapy, The Eighth Affiliated Hospital, Sun Yat-sen University, No. 3025 Shennan Zhong Road, Shenzhen, 518033 Guangdong China

**Keywords:** Chemoresistance, ITGA10, Osteosarcoma, PI3K/AKT pathway, ZIP10/SLC39A10

## Abstract

**Background:**

The zinc transporters Zrt- and Irt-related protein (ZIP/SLC39) are overexpressed in human tumors and correlate with poor prognosis; however, their contributions to carcinogenesis and chemoresistance in osteosarcoma (OS) remain unclear.

**Methods:**

We collected 64 OS patient tissues with (*n* = 12) or without (*n* = 52) chemotherapy. The expression levels of ZIP10 were measured by immunohistochemistry and applied to prognostic analysis. ZIP10 was knocked down or overexpressed in OS cell lines to explore its effect on proliferation and chemoresistance. RNA sequencing, quantitative real-time PCR, and western blotting analysis were performed to explore ZIP10-regulated downstream target genes. A xenograft mouse model was established to evaluate the mechanisms by which ZIP10 modulates chemoresistance in OS cells.

**Results:**

The expression of ZIP10 was significantly induced by chemotherapy and highly associated with the clinical outcomes of OS. Knockdown of ZIP10 suppressed OS cell proliferation and chemoresistance. In addition, ZIP10 promoted Zn content-induced cAMP-response element binding protein (CREB) phosphorylation and activation, which are required for integrin α10 (ITGA10) transcription and ITGA10-mediated PI3K/AKT pathway activation. Importantly, ITGA10 stimulated PI3K/AKT signaling but not the classical FAK or SRC pathway. Moreover, overexpression of ZIP10 promoted ITGA10 expression and conferred chemoresistance. Treatment with the CREB inhibitor 666–15 or the PI3K/AKT inhibitor GSK690693 impaired tumor chemoresistance in ZIP10-overexpressing cells. Finally, a xenograft mouse model established by subcutaneous injection of 143B cells confirmed that ZIP10 mediates chemotherapy resistance in OS cells via the ZIP10-ITGA10-PI3K/AKT axis.

**Conclusions:**

We demonstrate that ZIP10 drives OS proliferation and chemoresistance through ITGA10-mediated activation of the PI3K/AKT pathway, which might serve as a target for OS treatment.

**Supplementary Information:**

The online version contains supplementary material available at 10.1186/s13046-021-02146-8.

## Background

Osteosarcoma (OS) is the most common primary malignant bone tumor in children and adolescents [1]. For more than 30 years, chemotherapy has been considered an indispensable approach for treating advanced OS [2], and accordingly, neoadjuvant chemotherapy has improved the 5-year overall survival rate of OS from less than 20% to more than 60% [3]. Nonetheless, chemoresistance is currently a key factor leading to OS treatment failure [4], and there is an urgent need to identify new targets to reverse chemoresistance and develop more effective strategies for treatment.

Among the zinc (Zn) transporters located in the cytomembrane are members of the Znt/SLC30 and ZIP/SLC39 families [5]. ZIP/SLC39 family takes up Zn from the extracellular environment, whereas Znt/SLC30 family is responsible for Zn efflux. In recent years, increasing evidence has shown that the ZIP/SLC39 family is involved in regulating tumor progression [6]. For example, ZIP4 is highly expressed in pancreatic cancer and promotes proliferation and chemoresistance [7–9]. In OS, however, the role of Zn transporters has not been well explored. A study involving the OS cell line Saos-2 showed that ZIP10 is upregulated under conditions of Zn deficiency [10], suggesting that ZIP10 may be an important transporter for OS cells to maintain intracellular Zn levels. As some evidence suggests chemoprotective properties for Zn in cancers, the relationship between ZIP10 and chemoresistance in OS cells warrants attention.

Integrin α10 (ITGA10), one of the α subunits of type II collagen receptor integrins (α1β1, α2β1, and α10β1), was first isolated from chondrocytes [11]. ITGA10 exhibits a restricted tissue expression pattern and is most abundant in cartilage-containing tissues [12]. Moreover, gene knockout studies have shown that integrin α10β1 is essential for the formation of growth plates during bone development [13]. Although recent studies have suggested that ITGA10 may be involved in regulating tumor progression [14, 15], the expression and role of ITGA10 in OS cells have not yet been reported.

In our study, we found that ZIP10 is highly expressed in OS and is related to the clinicopathologic features of OS patients. ZIP10 activates CREB and thus promotes the expression of ITGA10 by increasing the intracellular Zn content. ITGA10 is a survival-related gene in OS that promotes cell proliferation and chemoresistance by activating PI3K/AKT signaling. Knockdown of ZIP10 significantly reduces the expression of ITGA10 and PI3K/AKT pathway activation. This study elucidates the mechanism by which ZIP10 is involved in cell proliferation and chemoresistance in OS, highlighting a promising approach for ZIP10-targeted therapy in OS treatment.

## Methods

### Specimen collection

OS tissues were obtained from 64 OS patients at the Eighth Affiliated Hospital, Sun Yat-sen University, Guangdong, China. Of these patients, 52 did not undergo chemotherapy, and 12 underwent chemotherapy (AP, doxorubicin 25 mg/m^2^, cisplatin 100 mg/m^2^). All specimens were frozen and maintained in liquid nitrogen.

### Cell lines and cell culture

Human OS cell lines (Saos2, U2OS and 143B) were obtained from the American Type Culture Collection (ATCC) Cell Bank and cultured according to the instructions from the ATCC. Starting with the 143B osteosarcoma cell line, the cisplatin-resistant variant was obtained by exposing the parental line to stepwise-increased cisplatin concentrations. This continuous exposure resulted in cells resistant to 3 μM cisplatin (143BR). Mesenchymal stem cells (MSCs) from healthy donors were isolated and cultured as previously described [16]. For stimulation of OS cells, ZnSO_4_ (15 μM; Sigma-Aldrich), Zn chelator TPEN (3 μM; TargetMol), cisplatin (0.5 μM; Sigma-Aldrich), CREB inhibitor 666–15 (0.1 μM; MCE), AKT activator SC79 (5 μM; MCE) and AKT inhibitor GSK690693 (10 μM; MCE) were added to the medium.

### RNA extraction and quantitative real-time PCR (qRT-PCR)

Total RNA was extracted from OS cells using TRIzol reagent (Invitrogen) and then used for cDNA synthesis as detailed by the manufacturer (TaKaRa). Quantitative analyses were carried out using SYBR Premix Ex Taq II reagents (TaKaRa), with ACTB as the internal reference. Relative expression was calculated based on the 2^-ΔCt^ method. The primer sequences are listed in Table S1.

### Protein preparation and western blotting (WB)

Total protein extracts were obtained for protein separation by 10% SDS-PAGE, followed by protein transfer onto PVDF membranes. After treatment with 5% nonfat milk, the membranes were probed with diluted primary antibodies (1:1000) against ACTB (CST; #4970), ZIP10 (SAB; #24824), AKT (CST; #4691), p-AKT (CST; #4060), ERK (CST; #4695), p-ERK (CST; #9101), JNK (CST; #9252), p-JNK (CST; #4671), C-PARP (CST; #5625), C-caspase-3 (CST; #9661), ITGA10 (SAB; #44824), PAK (CST; #2602), p-PAK (CST; #2606), p-FAK (CST; #8556), p-SRC (CST; #12432), CREB (CST; #12432), and p-CREB (CST; #9198) overnight at 4 °C. The membranes were then washed in TBST, followed by incubation with a diluted HRP-labeled secondary antibody (1:5000; CST; #7074 and #7076) for 2 h at room temperature. Signals were examined using an ECL detection system (Bio-Rad). Data were analyzed by ImageJ (version 1.8.0).

### Cell Counting Kit-8 (CCK-8) assay

The CCK-8 assay was used to assess cell proliferation. OS cells were seeded into 96-well plates at a density of 1 × 10^4^ cells/well and incubated for 24 h at 37 °C in 5% CO_2_. After 0, 1, 2, 3, and 4 days of cultivation, 10 μL CCK-8 reagent was added to each well followed by culture for 2 h at 37 °C in 5% CO_2_. Absorbance at 450 nm was measured using a microplate reader (Bio-Rad).

### EdU staining assay

Cell proliferation was monitored by EdU staining reagent (Beyotime) following the manufacturer’s instructions. Nuclear detection was carried out using Hoechst 33342 staining, after which the cells were observed by confocal microscopy (Nikon C2).

### Plate colony formation assay

Cells were treated with/without cisplatin for 48 h, seeded into 6-well plates (5 × 10^2^/well) and maintained at 37 °C in a 5% CO_2_ incubator for 2 weeks. Colonies were fixed with 4% paraformaldehyde and stained with 0.5% crystal violet, and their numbers were counted under a microscope. Three independent experiments were performed in triplicate.

### Flow cytometry

OS cells were seeded into 6-well plates. After culturing for 12 h, 0.5 μM cisplatin was added to each well for 3 days. After treatment, total cells were harvested and suspended at 1 × 10^6^ cells/mL; 5 μL Annexin V and 7-aminoactinomycin D (7-AAD) staining solution was added to 300 μL of the cell suspension. After incubation for 15 min at room temperature in the dark, the stained cells were assayed and quantified using a FACSort Flow Cytometer.

### Lentiviral infection and transient transfection

Briefly, to generate ZIP10-knockdown and ZIP10-overexpressing cells, pLKO.1-shZIP10 and pGC-FU-ZIP10 lentivirus plasmids were cotransfected into 293 T cells with psPAX2 and pMD2.G. Viral supernatant fractions were collected at 48 h after transfection followed by infection into Saos2 or 143B cells together with 6 μg/mL polybrene. After overnight infection, the cells were incubated in medium containing 2 μg/mL puromycin for 3 days. To knock down the expression of ITGA10 by siRNA transfection, OS cells in 6-well plates were transiently transfected with 150 pmol siRNA with Lipofectamine 3000 (Invitrogen; #11668–019) for 6 h following the manufacturer’s instructions. The target sequences are as follows: shZIP10#1: 5′-GCG TGA TCT TGG TTC CTA T-3′; shZIP10#2: 5′- GCA TTA GCT GTA GGA ACA A-3′; siITGA10: 5′-CCT GAG AGA AAT TAG AAC T-3′; NC: 5′-TTC TCC GAA CGT GTC ACG T − 3′.

### Zn concentration determination

OS cells (5 × 10^4^/well) were precultured overnight in 96-well plates, incubated for 1 h in PBS containing 1 μM FluoZin-3 AM (Life Technologies) and then washed three times with PBS. Fluorescence intensity (494 mm excitation/516 nm emission) was measured using a microplate reader (SpectraMax; Molecular Devices).

### Luciferase reporter assay

ITGA10-CRE was cloned into the pGL3-basic luciferase reporter plasmid. OS cells (2.5 × 10^4^ cells per well) were seeded in triplicate in 24-well plates. After culture for 12 h, the cells were transfected with 200 ng of ITGA10-CRE-luciferase-reporter plasmids. Each transfection included the same amount of Renilla luciferase plasmid, which was used to standardize the transfection efficiency. After incubation for 24 h, PBS, ZnSO4 or 666–15 was added to the medium and incubated for another 24 h. Firefly and Renilla signals were measured using a Dual Luciferase Reporter Assay kit (Promega), and the results are presented as the increase in activation over reporter alone.

### Chromatin immunoprecipitation (ChIP) assay

ChIP assays were performed according to the manufacturer’s instructions for the SimpleChIP enzymatic ChIP kit (CST). Briefly, a ChIP assay was performed using protein A/G agarose and an anti-p-CREB antibody. The immunoprecipitated DNA was used to amplify DNA fragments via PCR with specific primers. The primer sequences are listed in Table S2.

### In vivo tumor growth assay

Xenograft tumors were generated by subcutaneous injection of 143B cells (2 × 10^6^) into the flanks of 6-week-old athymic nude mice (*n* = 6/group). Tumors were measured using calipers every 3 days. For drug treatment, when the average tumor volume reached approximately 100 mm^3^, the mice were administered cisplatin (3 mg/kg/2 days), 666–15 (10 mg/kg/day) or GSK690693 (30 mg/kg/day) by intraperitoneal injection. The tumor volume was calculated according to the following formula: tumor volume (mm^3^) = length × width × width/2. Mice were euthanized at 30 days after cell injection to obtain tumor weights.

### Immunohistochemistry (IHC) staining

Tumor tissues obtained from OS patients or xenografts were embedded in paraffin and subjected to IHC staining with specific antibodies. The immunoreactions were evaluated independently by two pathologists. Briefly, the percentage of positive cells was scored as follows: 0, no positive cells; 1, ≤10% positive cells; 2, 10–50% positive cells; and 3, > 50% positive cells. Staining intensity was scored as follows: 0, no staining; 1, weak staining; 2, moderate staining; and 3, dark staining. The comprehensive score (0, 1, 2, 3, 4, 6, 9) = staining percentage × intensity. ZIP10 expression ≤2 indicates a low level, whereas > 2 indicates a high level.

### RNA sequencing (RNAseq)

RNA sequencing of ZIP10 knockdown cells was performed by BGI Tech based on DNBSEQ. In total, 6.8 GB clean data per sample were collected for RNAseq, and the clean reads were aligned to the human genome GRCh38 (Hg38).

### Statistical analysis

All assays were performed in biological triplicate, and the experimental results are given as the means ± standard deviation (SD) after processing using GraphPad PRISM 6. Group differences were estimated by the t test or one-way ANOVA, with *P* < 0.05 indicating statistical significance.

## Results

### ZIP10 promotes proliferation and chemoresistance in osteosarcoma

**Fig. 1:**
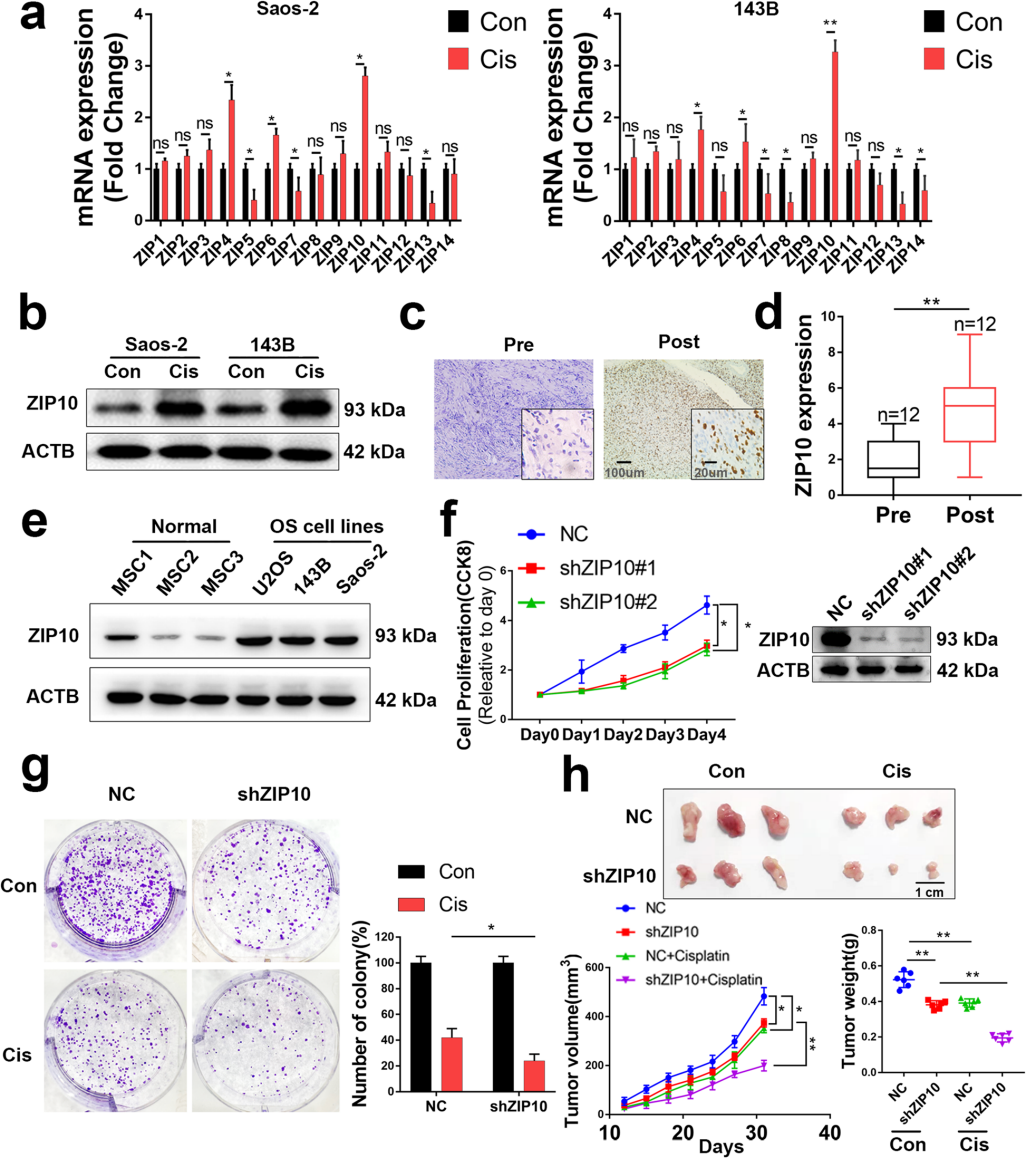
ZIP10 is required for osteosarcoma proliferation and chemoresistance. **a** qRT-PCR results of zinc transporter ZIP/SLC39 family expression in Saos-2 and 143B osteosarcoma (OS) cell lines with or without cisplatin treatment. **b** WB results of ZIP10 expression in 2 cell lines. **c** Representative IHC staining images of OS specimens with (post) or without (pre) chemotherapy. Scale bar, 100 μm. **d** ZIP10 expression level of OS specimens. **e** An analysis was performed to examine ZIP10 expression in human OS cell lines and normal mesenchymal stem cells (MSCs). **f** Left, CCK-8 assay was performed to determine the cell proliferation of negative control (NC) and stable ZIP10-knockdown (shZIP10) cell lines; right, the efficiency of shRNA targeting ZIP10 was determined by WB analysis. **g** Colony formation activity of 143B cells with/without cisplatin (Cis) treatment. **h** Photographed xenograft tumors (top), average tumor volume (bottom, left) and average tumor weight (bottom, right) of NC and shZIP20 xenografts with/without cisplatin treatment. Scale bar = 1 cm. *N* = 6 mice per group. Data are means ± SEM; *P < 0.05, **P < 0.01

Because Zn transporters ZIP/SLC39 have been implicated in tumor progression, we explored their role in OS chemoresistance. To determine whether ZIP/SLC39 expression was induced by cisplatin, we first treated the OS cell lines 143B and Saos-2 with 0.5 μM cisplatin for 24 h, a condition few cells underwent apoptosis (Fig. S1), excluding the effect of cell subpopulation selection by cisplatin. Then, we extracted total RNA for ZIP/SLC39 expression analysis. The results showed that ZIP10 expression in cisplatin-treated OS cells was nearly 3-fold that in untreated cells (Fig. 1a). Elevated ZIP10 expression (nearly 2.5-fold) in cisplatin-treated cells was also confirmed by WB (Fig. 1b and S3a). Both qRT-PCR and WB results indicated that ZIP10 expression increased during cisplatin treatment and reached a peak (nearly 4-fold in mRNA and nearly 3-fold in protein) at 72 h (Fig. S2). Accordingly, ZIP10 expression was higher in postchemotherapy patients (*n* = 12) than in prechemotherapy patients (*n* = 12; Fig. 1c, d), indicating that chemotherapy induces ZIP10 expression in OS. Moreover, ZIP10 was overexpressed in 3 OS cell lines tested compared to mesenchymal stem cells (MSCs) (Fig. 1e and S3b), which is a typical control cell line for OS cells. These results indicate that ZIP10 was highly induced by cisplatin treatment and might function in OS carcinogenesis and chemoresistance.

To explore the role of ZIP10 in OS, we generated stable ZIP10-knockdown OS cell lines (shZIP10 decreased by 80%). Knockdown of ZIP10 inhibited proliferation in 143B (Fig. 1f and S3c) and Saos-2 (Fig. S4a) cells and significantly enhanced cisplatin-induced suppression of colony formation (Fig. 1g and S4b). To determine the role of ZIP10 in the tumorigenesis and chemoresistance of OS in vivo, we established an athymic nude mouse model and found inhibition of tumor growth in the shZIP10 group (Fig. 1h). The average tumor weight of the vehicle-treated NC group was approximately 0.52 g, and the average tumor weight of the cisplatin-treated NC group was approximately 0.39 g, a 25% decrease compared to the vehicle-treated NC group. However, the average tumor weight of the cisplatin-treated shZIP10 group was approximately 0.20 g, which was 47% lower than that of the vehicle-treated shZIP10 group (0.38 g). Moreover, we generated stable ZIP10-overexpressing (oeZIP10) 143B cells. As shown in Fig. S5, ZIP10-overexpressing cells exhibited a higher proliferation rate and cisplatin resistance both in vitro and in vivo. These results indicate that ZIP10 positively regulates proliferation and chemoresistance in OS.

We next explored whether ZIP10 contributes to chemoresistance in drug-resistant cell lines. First, we exposed 143B cells to stepwise-increased cisplatin concentrations and generated cells resistant to 3 μM cisplatin (143BR). 143BR showed a proliferation rate and cellular morphology similar to those of 143B cells (Fig. S6a, b). In addition to resistance to cisplatin, 143BR also partially resisted adriamycin (ADR) and methotrexate (MTX) (Fig. S6c), indicating broad chemoresistance. Both qRT-PCR and WB results showed that ZIP10 expression increased by 100% in 143BR (Fig. S6d, e). By knocking down ZIP10 expression by 90% in 143BR cells, we found a decrease in the proliferation rate and cisplatin resistance both in vitro and in vivo (Fig. S7). These results indicated that high ZIP10 expression was associated with chemoresistance in 143BR. Overall, ZIP10 is overexpressed in OS and contributes to proliferation and chemoresistance.

### ZIP10 promotes OS proliferation and chemoresistance through the PI3K/AKT pathway

**Fig. 2:**
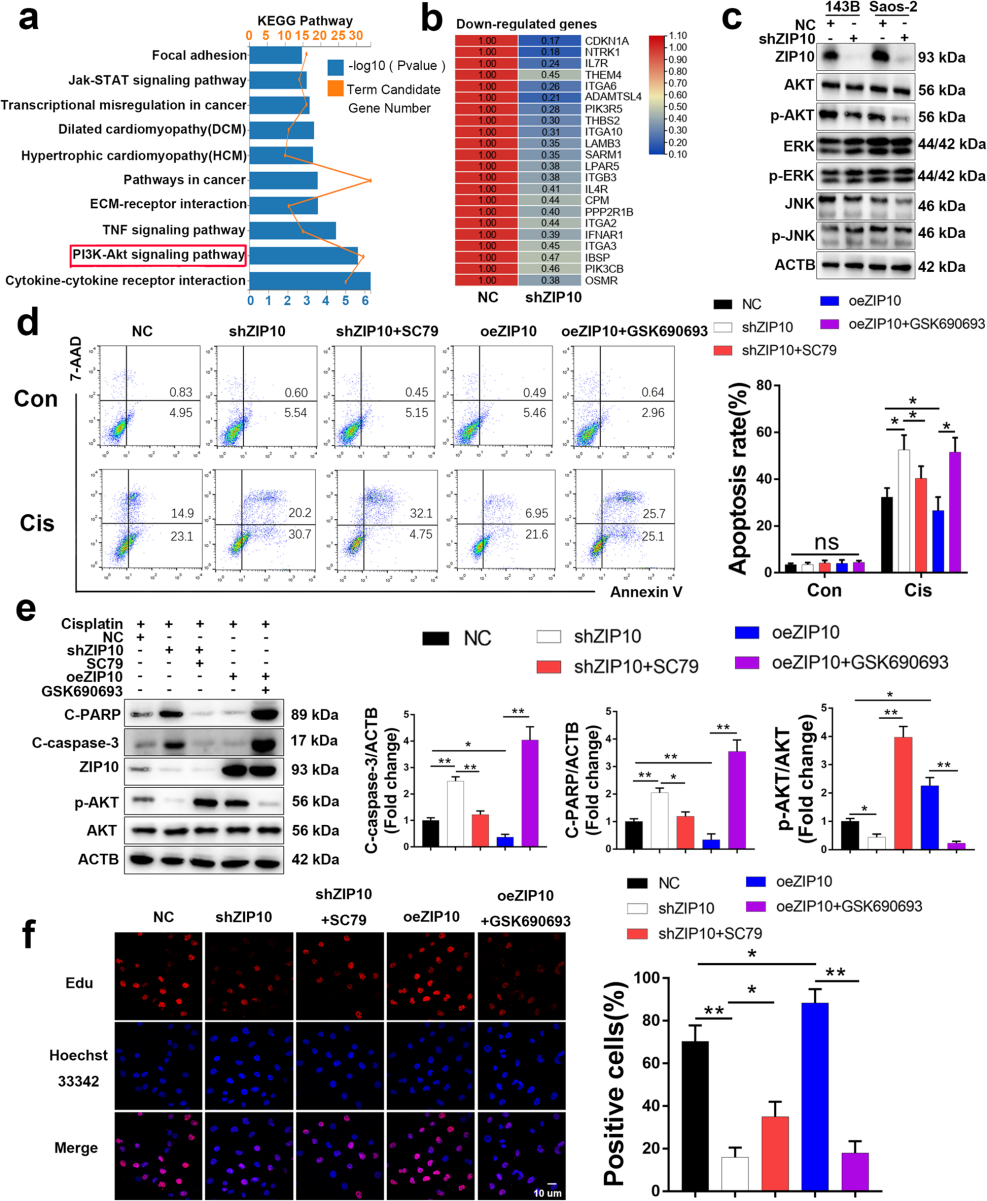
Knockdown of ZIP10 inhibits PI3K/AKT-mediated cell proliferation and chemoresistance in osteosarcoma. **a** KEGG pathway annotations of differentially expressed genes. The bar plot presents the enrichment scores (−log10[*P* value]) of the top 10 significantly enriched KEGG pathways. **b** Gene array analysis of NC and shZIP10 Saos-2 cells. Downregulated genes associated with the PI3K/AKT signaling pathway are shown. Rows represent individual genes; columns represent individual samples. Pseudocolors indicate transcript levels below (green), equal to (yellow) or above (red) the mean. The scale represents the relative gene expression (fold change between 0.1 and 1.1). **c** The expression levels of AKT, p-AKT, ERK, p-ERK, JNK and p-JNK were analyzed by WB using 143B and Saos-2 cells with/without ZIP10 knockdown. **d** ZIP10 knockdown (shZIP10), ZIP10 overexpression (oeZIP10), AKT activation (SC79) and AKT inhibitor (GSK690693) were applied to 143B cells treated with DMSO (Con) or cisplatin (Cis) for 72 h. Flow cytometry was conducted to determine apoptosis. **e** The whole-cell extract of 143B cells treated with cisplatin for 72 h was subjected to WB analysis. **f** EdU incorporation assay determination of the proliferation of 143B cells. Scale bar, 10 μm. Data are means ± SEM; *P < 0.05, **P < 0.01

To delineate the functional implications of ZIP10 in OS, we performed transcriptome sequencing to investigate expression changes in ZIP10-knockdown cells. According to Kyoto Encyclopedia of Genes and Genomes (KEGG) pathway analysis, differentially expressed genes were significantly enriched in gene sets involved in the PI3K/AKT pathway (Fig. 2a). Among them, 22 genes were downregulated (Fig. 2b) and 10 genes were upregulated (Fig. S8) in shZIP10 cells. These results indicate that PI3K/AKT signaling might be inhibited in shZIP10 OS cells. To examine this possibility, we analyzed several chemoresistance-associated signaling pathways in shZIP10 OS cells by WB and found that the protein level of p-AKT was decreased by 75–80% in shZIP10 OS cells but that those of p-ERK and p-JNK were not obviously changed (Fig. 2c and S9). These results indicate that ZIP10 contributes to the PI3K/AKT signaling pathway in OS cells.

To explore whether the ZIP10-mediated PI3K/AKT signaling pathway is associated with OS chemoresistance, we employed flow cytometry (Fig. 2d and S10a) and WB (Fig. 2e and S10b) to assess apoptosis induced by cisplatin. The results showed that the AKT activator SC79 rescued cisplatin-induced apoptosis in shZIP10 143B cells; in contrast, the AKT inhibitor GSK690693 attenuated the chemoresistance induced by ZIP10 overexpression (oeZIP10). Similar results of ZIP10-induced cell proliferation were observed after EdU incorporation (Fig. 2f and S10c). Consistent with these results, the protein level of p-AKT was increased by 200% in 143BR (Fig. S6e). All these results show that ZIP10 promotes cell proliferation and chemoresistance in OS through the PI3K/AKT pathway.

### ZIP10 activates the PI3K/AKT pathway by increasing ITGA10 expression

**Fig. 3:**
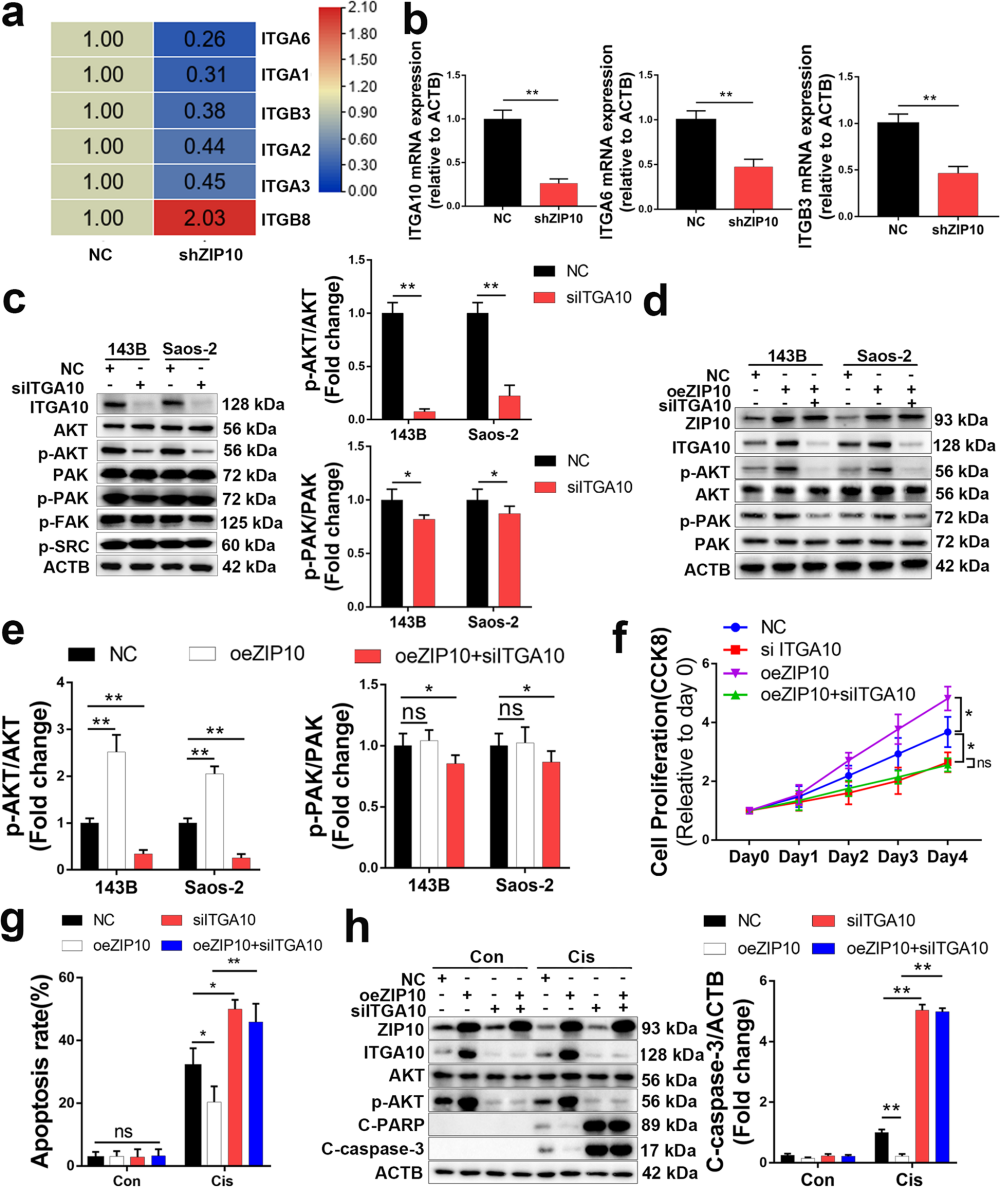
ZIP10 increases ITGA10 expression in osteosarcoma. **a** Gene array analysis of NC and shZIP10 Saos-2 cells. Up−/down-regulated genes associated with the integrin family are shown. **b** qRT-PCR analysis of integrins in Saos-2. **c** Expression levels of AKT, p-AKT, PAK, p-PAK, p-FAK and p-SRC were analyzed by WB using 143B and Saos-2 cells with/without ITGA10 knockdown. **d** WB analysis of AKT and PAK activation with/without ZIP10 overexpression in 143B cells. **e** Protein levels of p-AKT and p-PAK were quantified using ImageJ software. **f** A CCK-8 assay was performed to determine cell proliferation. **g** Flow cytometry was conducted to determine apoptosis. Apoptosis rates were quantified. **h** The whole-cell extract of 143B cells treated with/without cisplatin (Cis) for 72 h was subjected to WB analysis. Data are means ± SEM; *P < 0.05, **P < 0.01

We next explored how ZIP10 activates the PI3K/AKT pathway in OS. We noticed that the expression level of several integrins upstream of the PI3K/AKT pathway was altered in shZIP10 cells (Fig. 3a), which was confirmed by qRT-PCR (Fig. 3b and S11). Among these genes, ITGA10 had a nearly 70% decrease in shZIP10 cells. A previous study showed that ITGA10 activates the RAC/PAK and AKT pathways in myxofibrosarcoma [14], and we thus evaluated the expression of these signaling molecules in OS cells with/without ITGA10 knockdown (ITGA10 siRNA; siITGA10). Knockdown of ITGA10 significantly decreased p-AKT levels by 80–90% while slightly decreasing p-PAK levels but did not change p-FAK and p-SRC levels (Fig. 3c). Similarly, overexpression of ZIP10 increased the p-AKT level by 100–150%, which was attenuated by siITGA10 (Fig. 3d, e). We therefore hypothesize that ZIP10 activates AKT through ITGA10. We next performed CCK-8 assays (Fig. 3f), flow cytometry (Fig. 3g and S12) and WB (Fig. 3h) to further explore whether the ZIP10-ITGA10-p-AKT axis accounts for the observed proliferation and chemoresistance in oeZIP10 OS. The results showed that knockdown of ITGA10 attenuated oeZIP10-induced proliferation and chemoresistance in OS. Consistently, the expression of ITGA10 was increased by 200% in 143BR cells (Fig. S6d, e). Taken together, these results support that ZIP10 activates the PI3K/AKT pathway by promoting ITGA10 expression.

### ZIP10 increases ITGA10 expression by activating CREB

**Fig. 4:**
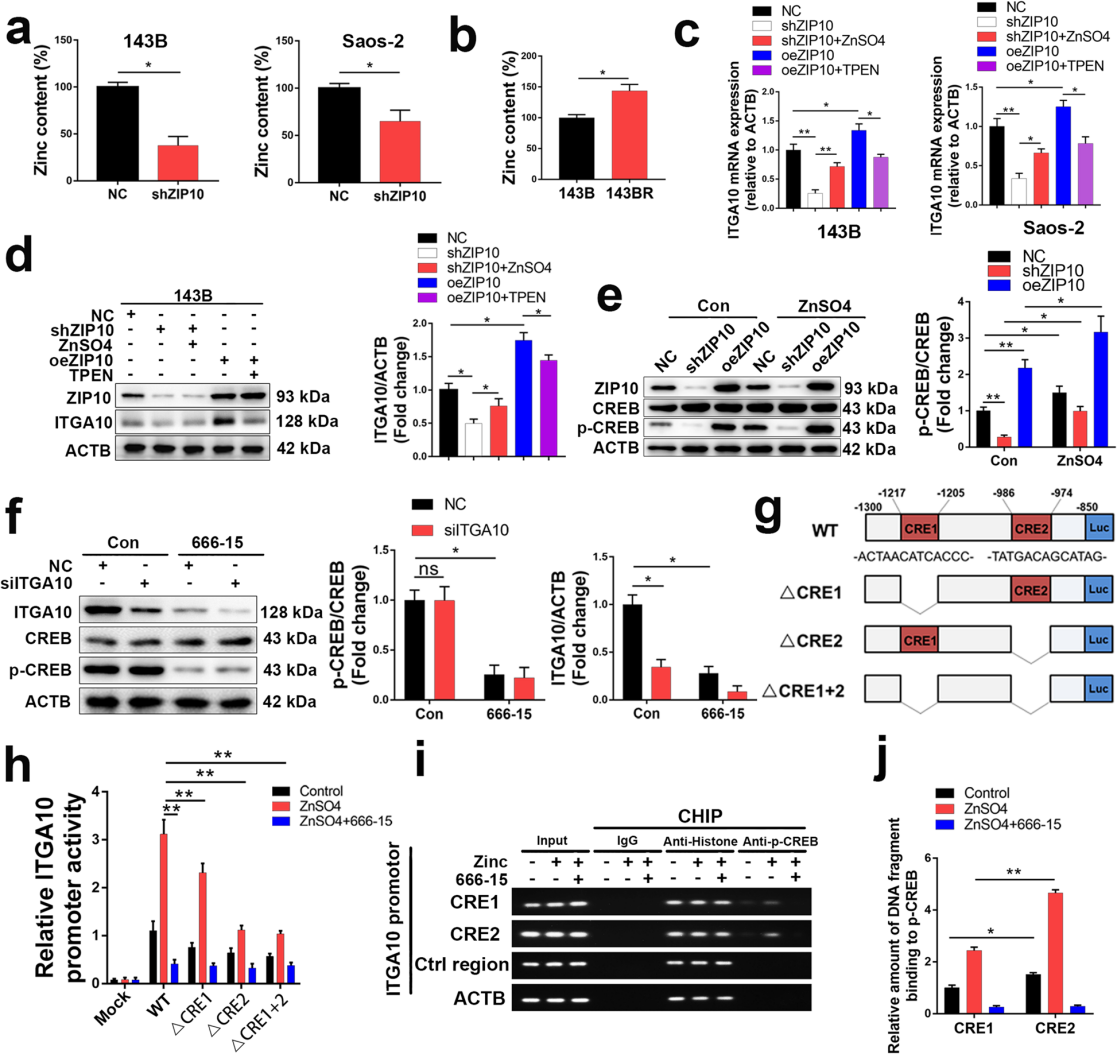
ZIP10 increases CREB phosphorylation, leading to upregulation of ITGA10 transcription. **a**, **b** Zn concentrations were analyzed in OS cells with/without ZIP10 knockdown using FluoZin-3 AM. **c**, **d** ITGA10 mRNA (**c**) and protein (**d**) levels were analyzed in OS cells treated with Zn supplementation (ZnSO_4_) or Zn chelator (TPEN). **e** WB analysis of CREB and p-CREB expression in 143B cells treated with/without ZnSO_4_. **f** WB analysis of p-CREB in 143B cells with/without ITGA10 knockdown. WB analysis of ITGA10 in 143B cells with/without CREB inhibition (666–15). **g** Schematic diagram showing the promoter structures of luciferase constructs: the wild-type ITGA10 promoter (WT), deletion mutants lacking CRE1 (△CRE1), CRE2 (△CRE2), or both (△CRE1 + 2). The CRE1 and CRE2 motif sequences are indicated. TSS, transcription start site. LUC, luciferase. **h** 143B cells were transfected with plasmids encoding *ITGA10*(− 1300/− 800)-Luc WT, △CRE1, △CRE2 or △CRE1 + 2. Then, OS cells were treated with Zn supplementation (ZnSO_4_) or CREB inhibitor (666–15). Luciferase levels were measured in extracts after 48 h. **i** ChIP of putative CREB response elements (CRE 1 and 2) within the ITGA10 promoter region determined by PCR. The promoter’s non-CRE region (Ctrl motif) and the β-actin region were used as negative controls; histone antibody or rabbit IgG were assay controls. **j** ChIp results confirmed by qRT-PCR. Data are means ± SEM; *P < 0.05, **P < 0.01

Because Zn transporters ZIP/SLC39 primarily transport Zn from the extracellular to intracellular space, many studies have shown that knockdown of ZIP decreases Zn concentrations in cells [17]. In this study, we confirmed a lower Zn content in shZIP10 OS cells (Fig. 4a) and a higher Zn content in 143BR (Fig. 4b). Intracellular Zn levels were shown to regulate cell proliferation and survival in cancers [6]. We treated OS cells with 0, 5, 10, 15, 30, 50, 100, and 200 μM ZnSO_4_ and 0, 1, 3, 5, 10, and 20 μM TPEN (a Zn chelator) to evaluate the role of Zn in proliferation and chemoresistance. The results showed that both 143B and Saos-2 proliferation increased with increasing ZnSO_4_ concentrations lower than 15 μM but decreased with increasing ZnSO_4_ concentrations higher than 30 μM (Fig. S13a). However, 143B and Saos-2 proliferation decreased with increasing TPEN concentrations (Fig. S13b). Since the human serum Zn concentration is approximately 12–20 μM [18], a decrease in Zn levels might inhibit the proliferation of OS in vivo. Moreover, apoptosis assays showed that 15 μM ZnSO4 increased the chemoresistance of OS cells, while 3 μM TPEN inhibited chemoresistance (Fig. S13c). All these results indicated that an appropriate increase in cellular Zn levels can lead to increased chemoresistance of OS cells.

We next explored whether ITGA10 expression is regulated by the Zn content in OS cells. As revealed by qRT-PCR (Fig. 4c) and WB (Fig. 4d), Zn supplementation rescued the decrease in ITGA10 expression in shZIP10 143B cells, whereas TPEN attenuated the increase in ITGA10 expression induced by ZIP10 overexpression. These results demonstrate that ITGA10 expression is regulated by Zn content in OS.

As previous studies have reported that the Zn content regulates cyclic AMP response element-binding protein (CREB) activation in cancer cells [19, 20], we next explored p-CREB/CREB levels in OS cells. Knockdown of ZIP10 decreased the p-CREB level by 80% in 143B cells, and Zn supplementation rescued this decrease in p-CREB (Fig. 4e). ITGA10 expression and CREB activation are both regulated by the Zn content, and we thus explored their relationship. As shown in Fig. 4f, the CREB inhibitor 666–15 inhibited ITGA10 expression by 65%, whereas knockdown of ITGA10 did not alter the p-CREB level. These results indicate that CREB is upstream of ITGA10. CREB is a basic leucine zipper (bZIP) transcription factor that recognizes the palindromic cAMP response element (CRE) motif 5′-TGA CGT CA-3′ [21]. We used JASPAR to search the ITGA10 promoter, i.e., the − 2000 bp to 0 bp sequence upstream of the transcription start site (TSS), and found candidate CRE sequences (CRE1 and CRE2). To analyze ITGA10 promoter motifs required for Zn-mediated expression, we assessed ITGA10 promoter luciferase constructs with deletions in the CRE1 and/or CRE2 motifs (Fig. 4g). Zn supplement-induced ITGA10 promoter activation was substantially reduced by deletion of the CRE2 motif, although CRE1 deletion decreased ITGA10 promoter activity to a lesser extent (Fig. 4h). Deletion of both CRE motifs repressed ITGA10 promoter activity comparable to CRE2 deletion alone. Direct binding of the ITGA10 promoter by CREB was also monitored by anti-p-CREB ChIP, followed by polymerase chain reaction (PCR) and qRT-PCR for the CRE motifs in the ITGA10 promoter. ChIP revealed enriched p-CREB binding within regions of the putative CRE motifs predicted by JASPAR. Moreover, the binding of p-CREB to CRE1 and CRE2 was increased by ZnSO4 treatment, with CRE2 showing more enrichment than CRE1 (Fig. 4i, j). These results indicate that CREB mediates ITGA10 transcription. Taken together our findings show that ZIP10 increases CREB phosphorylation, leading to upregulation of ITGA10 transcription.

### The ZIP10-ITGA10-p-AKT axis confers OS cell chemoresistance in vivo

To investigate the role of the ZIP10-ITGA10-p-AKT axis in the regulation of OS cell proliferation and sensitivity to chemotherapy in vivo, we performed xenograft tumor experiments using 143B and 143BR cells.

**Fig. 5:**
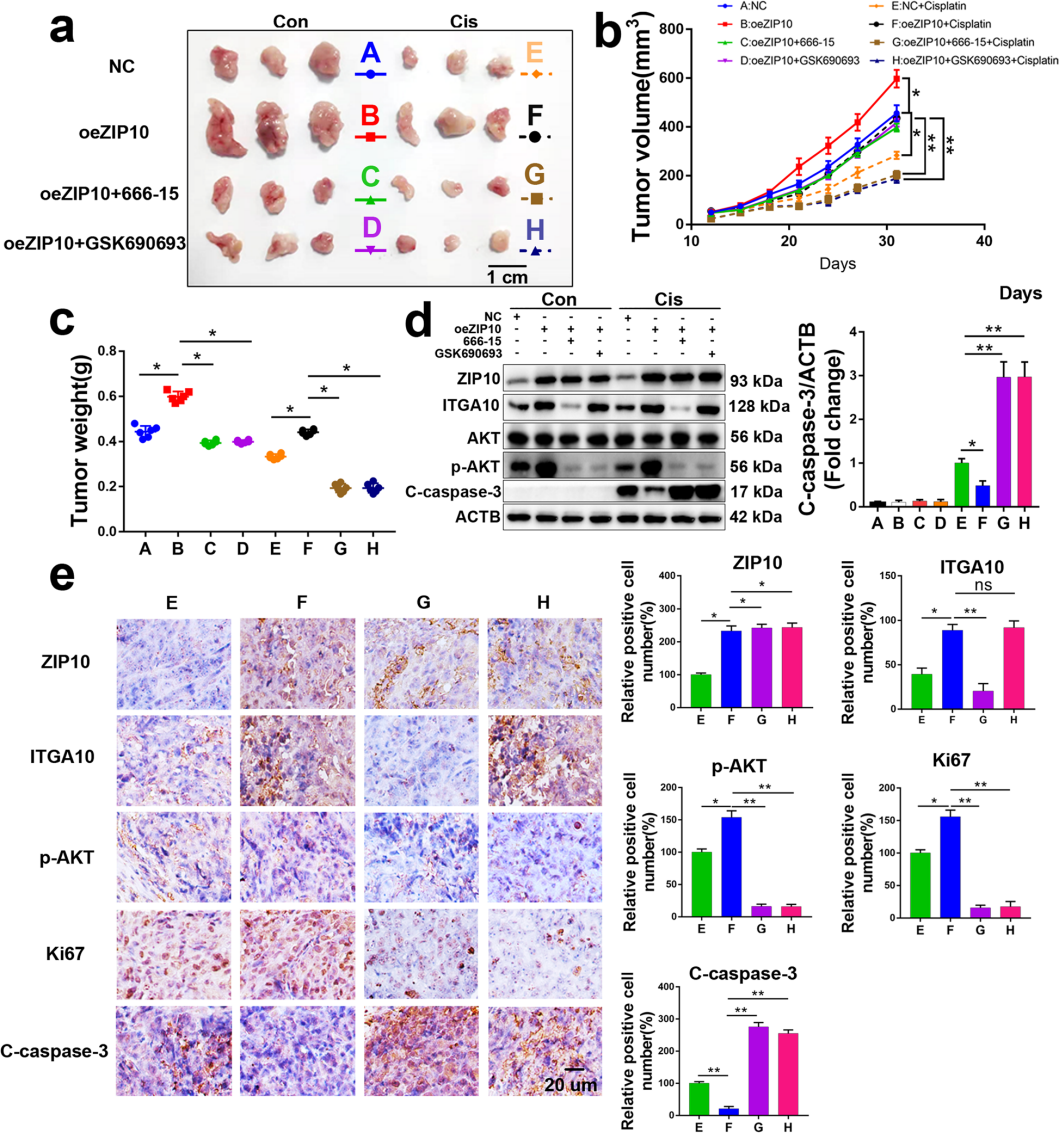
ZIP10-ITGA10-p-AKT signaling is required for the maintenance of cisplatin resistance in vivo. **a**-**c** Photographed xenograft tumors (**a**), average tumor volume (**b**) and tumor weight (**c**) of 143B-NC, 143B-oeZIP10, 143B-oeZIP10 + 666–15 and 143B-oeZIP10 + GSK690693 xenografts treated with vehicle (Con) or cisplatin (Cis). Scale bar, 1 cm. *N* = 6 mice per group. **d** WB analysis was performed to detect target proteins, as indicated in xenograft tissues. **e** IHC staining analysis of ZIP10, ITGA10, p-AKT, Ki67 and C-caspase-3 in xenograft tissues. Scale bar, 20 μm. Data are means ± SEM; *P < 0.05, **P < 0.01

In 143B cells, tumor growth and chemoresistance were promoted by ZIP10 overexpression, and this promotion was attenuated by the CREB inhibitor 666–15 or AKT inhibitor GSK690693 (Fig. 5a-c). Overexpression of ZIP10 increased the protein levels of ITGA10 and p-AKT; 666–15 reduced ITGA10 and p-AKT, and GSK690693 decreased p-AKT (Fig. 5d). Furthermore, IHC staining results indicated that overexpression of ZIP10 increased Ki67 and p-AKT expression in xenograft tissues with/without cisplatin treatment but that oeZIP10 + 666–15 or oeZIP10 + GSK690693 did not (Fig. 5e and S14). In addition, apoptosis, as analyzed by cleaved caspase 3 (C-caspase-3) staining, was dramatically decreased in cisplatin-treated oeZIP10 xenograft tissues.

For 143BR, knockdown of ZIP10 dramatically inhibited tumor growth and chemoresistance, and this inhibition was rescued by the AKT activator SC79 (Fig. S15a-c). Knockdown of ZIP10 decreased the protein levels of ITGA10 and p-AKT, and SC79 increased p-AKT (Fig. S15d). Furthermore, IHC staining results indicated that knockdown of ZIP10 increased C-caspase-3 levels in xenograft tissues with cisplatin treatment but that shZIP10 + SC79 did not (Fig. S15e).

Taken together, these findings suggest that the activated ZIP10-ITGA10-p-AKT axis is indispensable for cisplatin resistance in OS cells.

### Upregulation of ZIP10-ITGA10-p-AKT correlates with clinicopathologic features in OS patients

**Fig. 6:**
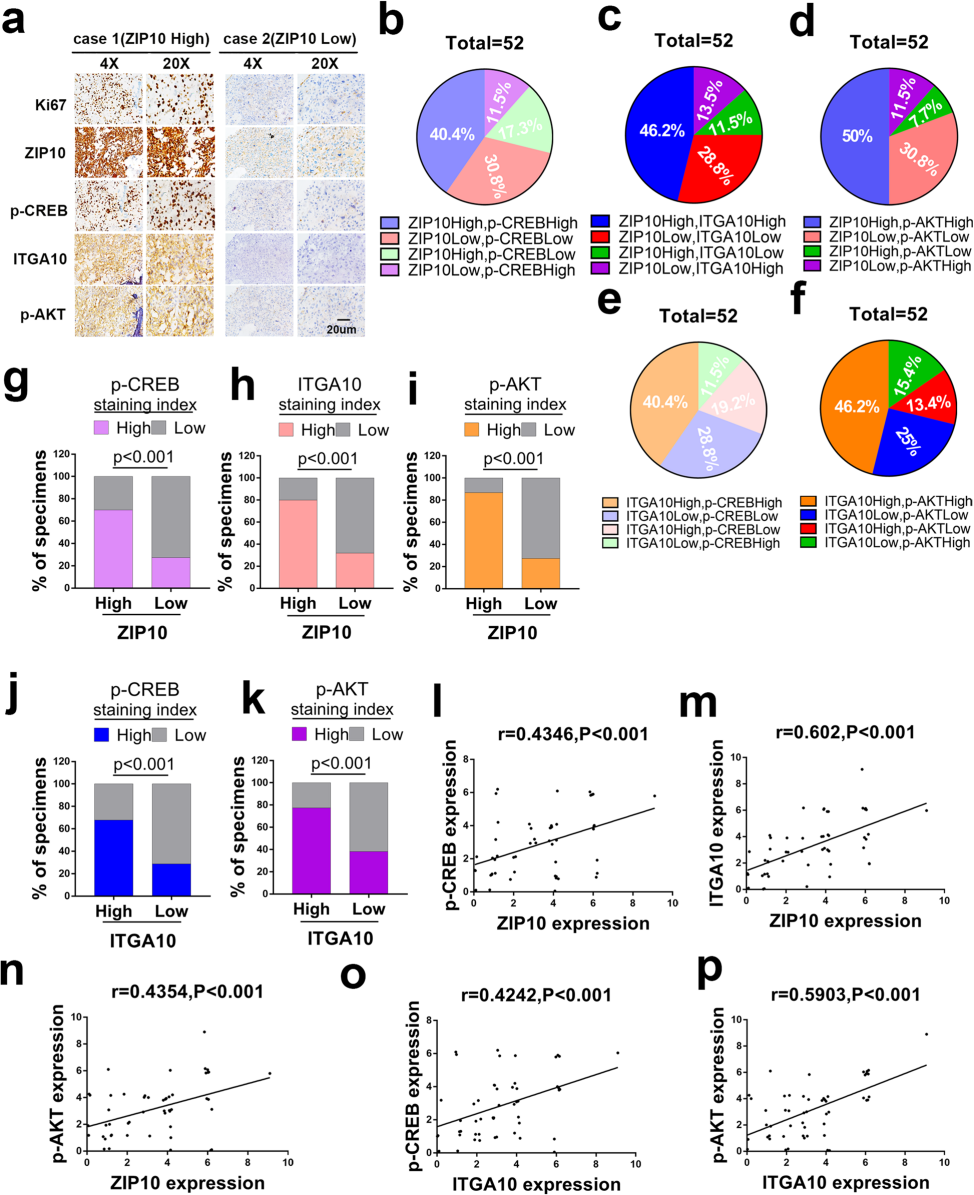
ZIP10 correlates positively with p-CREB, ITGA10 and p-AKT in osteosarcoma patients. **a** Representative cases from 52 OS specimens (without chemotherapy) were analyzed by IHC staining for Ki67, ZIP10, p-CREB, ITGA10 and p-AKT. Scale bar, 20 μm. **b**-**f** The expression of ZIP10 and p-CREB (**b**), ZIP10 and ITGA10 (**c**), ZIP10 and p-AKT (**d**), ITGA10 and p-CREB (**e**), and ITGA10 and p-AKT (**f**) was analyzed in 52 OS specimens. The relative proportions of protein expression are illustrated as a pie chart. **g**-**i** The percentage of samples displaying low or high ZIP10 expression compared to the expression levels of p-CREB (**g**), ITGA10 (**h**) and p-AKT (**i**). **j**, **k** The percentage of specimens displaying low or high ITGA10 expression compared to the expression levels of p-CREB (**j**) and p-AKT (**k**). **l**-**p** Scatterplot showing the positive correlation between ZIP10 and p-CREB (**l**), ZIP10 and ITGA10 (**m**), ZIP10 and p-AKT (**n**), ITGA10 and p-CREB (**o**), ITGA10 and p-AKT (**p**) expression in OS patients

To determine whether ZIP10, p-CREB, ITGA10 and p-AKT levels correlate in OS, we collected 52 patient tumor tissue sections without chemotherapy. The association between ZIP10 level and clinical features of patients, including gender, age, anatomical site, Enneking’s stage and AJCC stage, are summarized in Table S3. ZIP10 correlated positively with Enneking’s stage (*p* = 0.0238) and AJCC stage (*p* = 0.0111). Representative staining images with high or low levels of ZIP10, p-CREB, ITGA10 and p-AKT expression are shown in Fig. 6a. ZIP10, p-CREB, ITGA10 and p-AKT scores were evaluated by systematically analyzing the IHC staining results (Fig. 6b-f). Among 52 patients, 21 cases of high p-CREB levels were found for all 30 individuals with a high level of ZIP10 staining; 24 of 30 patients with a high level of ZIP10 exhibited an upregulated level of ITGA10 protein, and 26 of 30 patients with a high level of ZIP10 showed an upregulated level of p-AKT protein (Fig. 6g-i). Additionally, increased CREB and AKT activation was accompanied by ITGA10 overexpression (Fig. 6j, k). As expected, statistically significant positive correlations between ZIP10 and p-CREB (Fig. 6l), ZIP10 and ITGA10 (Fig. 6m), ZIP10 and p-AKT (Fig. 6n), ITGA10 and p-CREB (Fig. 6o) and ITGA10 and p-AKT (Fig. 6p) were observed. These findings suggest that ZIP10 leads to ITGA10-mediated AKT activation associated with clinicopathologic features and may contribute to tumorigenesis of OS.

## Discussion

**Fig. 7:**
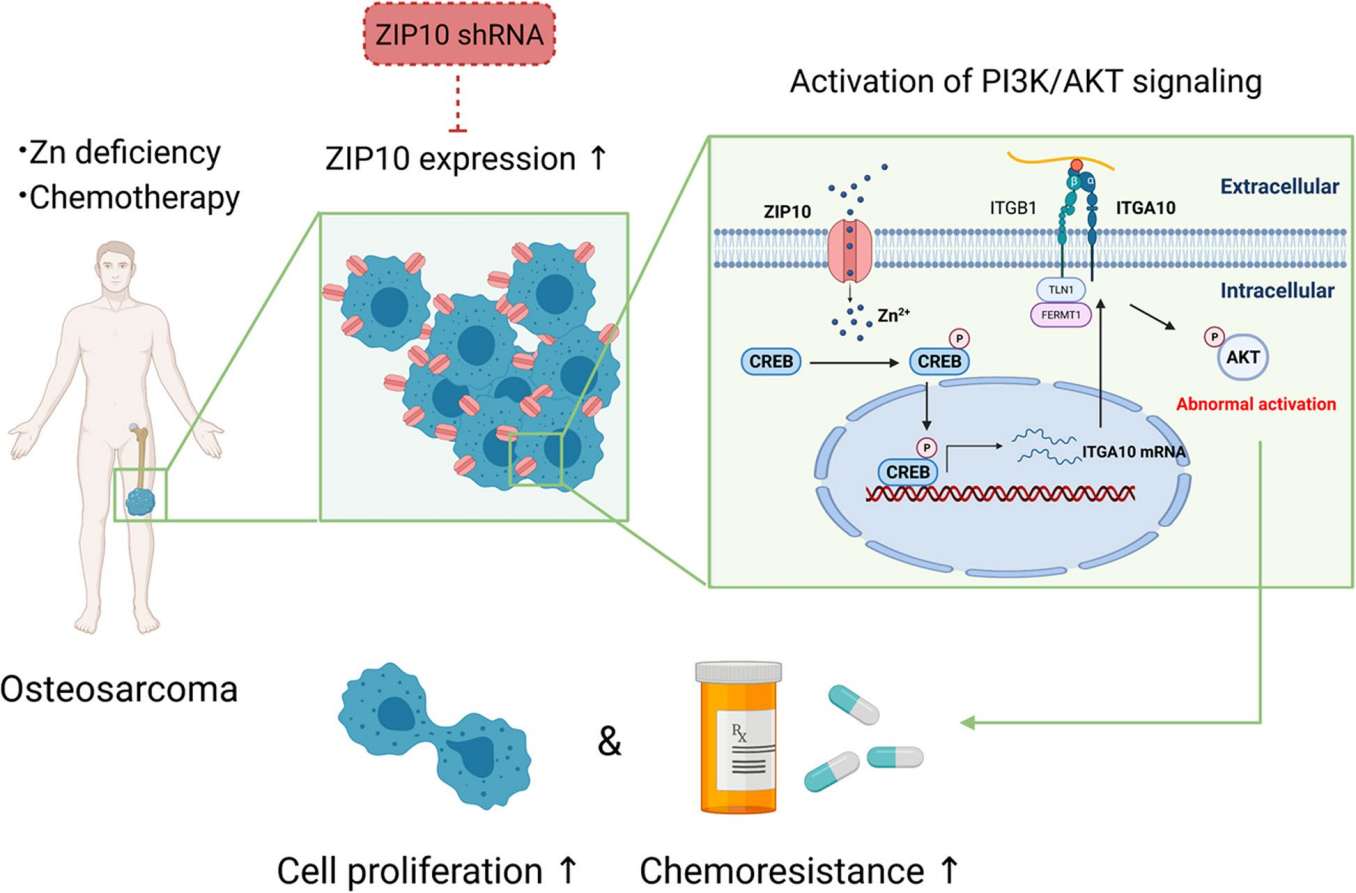
Proposed model of ZIP10 regulation of proliferation and chemoresistance in osteosarcoma. ZIP10 expression is elevated in OS, which might be a result of Zn deficiency and chemotherapy. ZIP10 increases Zn uptake and activates CREB and its binding to the ITGA10 promotor and thus promotes ITGA10 expression in OS cells. ITGA10 is an important membrane-anchored protein that confers OS cell proliferation and chemoresistance through activation of the PI3K/AKT signaling pathway. Targeting ZIP10 in OS might increase chemotherapy sensitization in OS

Zn is an important cofactor for many proteins and plays a vital role in DNA synthesis, enzyme activity and nucleic acid metabolism [6]. It is estimated that approximately 3000 human proteins contain “zinc finger” motifs [22], and Zn has recently been considered to be a “second messenger” [23]. The cellular concentration and temporal and spatial regulation of Zn drive extensive changes in cell fate through different signal transduction pathways. In our study, we show that ZIP10-mediated Zn absorption promotes cell proliferation and chemoresistance by activating the CREB-ITGA10-PI3K/AKT signaling cascade in OS (Fig. 7).

It has been proven that the PI3K/AKT pathway is involved in the chemoresistance process in a variety of tumors [24]. AKT-overexpressing cells displayed hyperexpression of antiapoptotic Bcl-xL and delayed activation of the p53 signaling pathway upon exposure to cisplatin or mitoxantrone [25]. In OS, the PI3K/AKT pathway is a commonly deregulated oncogenic signaling pathway, and chromosomal loss of PTEN, which antagonizes PI3K/AKT, is a frequent event [26]. In this study, we found that the PI3K/AKT pathway helps OS cells resist cisplatin-induced apoptosis. Elevated PI3K/AKT signaling in OS occurs not only due to a lack of PTEN inhibition but also due to increased ZIP10-mediated ITGA10 expression.

Many integrin members were shown to activate the PI3K/AKT pathway in previous studies [27]. As integrins lack intrinsic catalytic activity, they transmit signals through activation of integrin-associated proteins. Most integrins engage the actin network via talin and additional proteins, leading to integrin clustering and the ensuing activation of FAK and SRC. These processes link integrins to downstream signaling effectors, such as the PI3K/AKT, Ras-ERK and YAP/TAZ pathways. However, T. Okada et al. showed that ITGA10 activated PI3K/AKT signaling without activating the classical FAK or SRC pathway [14], which was also supported by our results. These results indicate that ITGA10 may represent a nonclassical means of inducing the signaling cascade. Furthermore, T. Okada et al. also showed that RICTOR directly binds to ITGA10 and transmits activation signals from collagen-binding ITGA10 to the PI3K/AKT pathway. However, they did not explore whether intracellular RICTOR binds to the cytomembrane-localized ITGA10 intracellular domain, which contains only 22 amino acids. In other words, RICTOR was still not fully proven to be a direct interactor of ITGA10. Intriguingly, in our study, we found that ITGA10 interacts with several receptor tyrosine kinases (data not shown). Whether ITGA10 activates the PI3K/AKT pathway by directly regulating these receptor tyrosine kinases remains a very interesting question.

Epidemiological evidence indicates that serum Zn levels are significantly reduced in most cancers, including OS, even though the Zn content of tumor tissues varies according to tumor type [6]. For example, the Zn content of tumor tissues is higher than that of normal tissues in breast cancer, lung cancer and bowel cancer, whereas it is lower than that of normal tissues in prostate cancer, pancreatic cancer and liver cancer [28, 29]. Furthermore, there are two studies describing conflicting results for tumor Zn content in OS. Gao K. et al. indicated that the Zn content in OS tissue is lower than that in adjacent tissues [30], whereas Rauwolf M. et al. found that the ratio of Zn count rate medians in mineralized tumor tissue was approximately 6 times higher than that in normal bone [31]. Overall, it is difficult to conclude differences in Zn content between OS and normal tissues due to the great difference between soft tissue and bone tissue in OS. Further studies to determine an accurate Zn content in different regions of OS tissue are needed.

Recently, many studies have pointed out that high concentrations of Zn may constitute a new treatment strategy for OS [32]. Indeed, high-concentration Zn therapy can exert antitumor activity on OS cells by inhibiting proliferation and invasion and promoting apoptosis and chemotherapy sensitization [30, 33]. However, in our research, we found that Zn treatment at a dose of 15 μM, similar to that of the human serum Zn concentration (12–20 μM) [18], increases CREB-mediated ITGA10 expression in OS, which might lead to chemotherapy resistance. On the other hand, decreasing the intracellular Zn content by ZIP10 knockdown impaired the proliferation and chemoresistance of OS cells. The above results suggest that an appropriate amount of intracellular Zn promotes OS cell proliferation and chemoresistance but that Zn levels that are too low or too high are inhibitory.

Previous studies have suggested that ZIP10 may be an important channel for Zn uptake in OS cells, as its expression is upregulated when the environment is deficient in Zn [10]. In our study, we confirmed the importance of ZIP10 in managing the Zn content in OS. Furthermore, we showed that ZIP10 plays a vital role in promoting cell proliferation and chemoresistance by activating the PI3K/AKT pathway in OS. These results suggest that targeting ZIP10 to inhibit Zn uptake may represent an effective treatment for OS.

Current studies show that CREB plays a significant role in promoting progression in a variety of tumors, while our results show that it also plays an important role in chemotherapy resistance in OS. As a result, CREB has become a very promising potential antitumor target [21]. However, it remains difficult to make progress using inhibitors targeting CREB due to side effects such as toxicity and off-target effects. Therefore, new strategies to inhibit CREB are needed. In our results, we found that ZIP10 in OS increased CREB activity through Zn absorption. CREB activity was significantly inhibited by the knockdown of ZIP10. As transport proteins are easier to target by small chemical compounds, ZIP10 might represent a new target to inhibit CREB activity.

## Conclusions

In conclusion, this study shows that ZIP10 plays a vital role in cell proliferation and chemoresistance by promoting CREB-mediated ITGA10 transcription and activation of PI3K/AKT signaling. This finding reveals that ZIP10 might serve as a target for OS treatment.

## Supplementary Information

**Additional file 1: Fig. S1.** Cisplatin induced OS cells apoptosis in a dose- and time-dependent manner. **Fig. S2.** qRT-PCR and WB analysis of ZIP10 expression in OS cells during cisplatin treatment. **Fig. S3.** Quantification of ZIP10 expression based on WB. **Fig. S4.** ZIP10 knockdown inhibits cell proliferation and chemoresistance in Saos-2 cells. **Fig. S5.** ZIP10 overexpression promotes cell proliferation and chemoresistance in 143B cells. **Fig. S6.** Chemoresistance evaluation and gene expression of the cisplatin-resistant variant 143BR. **Fig. S7.** ZIP10 knockdown inhibits cell proliferation and chemoresistance in 143BR cells. **Fig. S8.** Gene array analysis of NC and shZIP10 Saos-2 cells. **Fig. S9.** Quantification of signaling pathways based on WB. **Fig. S10.** Knockdown of ZIP10 inhibits PI3K/AKT-mediated cell proliferation and chemoresistance in Saos-2 cells. **Fig. S11.** qRT-PCR analysis of integrin expression in Saos-2 cells with/without ZIP10 knockdown. **Fig. S12.** Flow cytometry analysis of cisplatin-induced apoptosis in 143B cells with/without ZIP overexpression or ITGA10 knockdown. **Fig. S13.** The effect of Zn on the proliferation and chemoresistance of 143B cells and Saos-2 cells. **Fig. S14.** IHC staining analysis of Ki67, ZIP10, ITGA10, p-AKT and cleaved caspase 3 in xenograft tissues without cisplatin treatment. **Fig. S15.** The ZIP10-ITGA10-p-AKT signaling is required for cisplatin resistance in 143BR.

**Additional file 2: Table S1.** Primer sequences for qRT-PCR.

**Additional file 3: Table S2.** Primer sequences for ChIP assay.

**Additional file 4: Table S3.** Relationships between ZIP10 expression and clinical pathological characteristics of 52 patients (without chemotherapy) of osteosarcoma.

## Data Availability

The datasets supporting the conclusions of this article are available from the corresponding author on reasonable request.

## Abbreviations

RNA-seq: RNA sequencing
qRT-PCR: Quantitative real-time PCR
OS: Osteosarcoma
CREB: cAMP-response element binding protein
ITGA10: Integrin α10
ATCC: American Type Culture Collection
MSCs: Mesenchymal stem cells
cDNA: Complementary DNAs
ECL: Enhanced chemiluminescence
CCK-8: Cell Counting Kit 8
7-AAD: 7-aminoactinomycin D
ChIP: Chromatin immunoprecipitation
IHC: Immunohistochemistry
