# Supplementary material for: ZIP10 drives osteosarcoma proliferation and chemoresistance through ITGA10-mediated activation of the PI3K/AKT pathway
Source: J Exp Clin Cancer Res. 2021 Oct 27;40:340. doi: 10.1186/s13046-021-02146-8 (PMC8549349; doi:10.1186/s13046-021-02146-8)
Supplement: Supplementary file 1 — Additional file 1: Fig. S1. Cisplatin induced OS cells apoptosis in a dose- and time-dependent manner. Fig. S2. qRT-PCR and WB analysis of ZIP10 expression in OS cells during cisplatin treatment. Fig. S3. Quantification of ZIP10 expression based on WB. Fig. S4. ZIP10 knockdown inhibits cell proliferation and chemoresistance in Saos-2 cells. Fig. S5. ZIP10 overexpression promotes cell proliferation and chemoresistance in 143B cells. Fig. S6. Chemoresistance evaluation and gene expression of the cisplatin-resistant variant 143BR. Fig. S7. ZIP10 knockdown inhibits cell proliferation and chemoresistance in 143BR cells. Fig. S8. Gene array analysis of NC and shZIP10 Saos-2 cells. Fig. S9. Quantification of signaling pathways based on WB. Fig. S10. Knockdown of ZIP10 inhibits PI3K/AKT-mediated cell proliferation and chemoresistance in Saos-2 cells. Fig. S11. qRT-PCR analysis of integrin expression in Saos-2 cells with/without ZIP10 knockdown. Fig. S12. Flow cytometry analysis of cisplatin-induced apoptosis in 143B cells with/without ZIP overexpression or ITGA10 knockdown. Fig. S13. The effect of Zn on the proliferation and chemoresistance of 143B cells and Saos-2 cells. Fig. S14. IHC staining analysis of Ki67, ZIP10, ITGA10, p-AKT and cleaved caspase 3 in xenograft tissues without cisplatin treatment. Fig. S15. The ZIP10-ITGA10-p-AKT signaling is required for cisplatin resistance in 143BR. [file 13046_2021_2146_MOESM1_ESM.zip › Addiitional file 1.docx]

**Supplementary information**

**Fig. S1** Cisplatin induced OS cells apoptosis in a dose- and time-dependent manner. **a.** Flow cytometry analysis of apoptosis rate in 143B cells treated with cisplatin of different concentrations (0, 0.5, 1, 5, 10 μM) for 24 h. **b.** Flow cytometry analysis of apoptosis rate in 143B and Saos-2 cells treated with 0.5 μM cisplatin for 0, 24, 48, 72, 96 h. **c.** Flow cytometry analysis data show ZIP10 expression spectra of 143B cells and Saos-2 cells. Cells in a cell line contain different ZIP10 levels. A small subset of 143B cells were ZIP10 negative. Data are means ± SEM; *P<0.05, **P<0.01.

**Fig. S2** qRT-PCR and WB analysis of ZIP10 expression in OS cells during cisplatin treatment. **a, b.** qRT-PCR (**a**) and WB (**b**) analysis show that the ZIP10 expression level gradually increased with time of cisplatin treatment. Data are means ± SEM; *P<0.05, **P<0.01.

**Fig. S3** Quantification of ZIP10 expression based on WB. **a.** Quantification of ZIP10 expression in 2 cell lines treated with/without cisplatin (Cis). **b.** Quantification of ZIP10 expression in human OS cell lines and normal mesenchymal stem cells (MSCs). **c.** Quantification of ZIP10 expression with/without ZIP10 knockdown. Data are means ± SEM; *P<0.05, **P<0.01.

**Fig. S4** ZIP10 knockdown inhibits cell proliferation and chemoresistance in Saos-2 cells. **a**. Left, the CCK-8 assay was performed to determine the proliferation of negative control (NC) and stable ZIP10-knockdown (shZIP10) Saos-2 cells; right, the efficiency of shRNA targeting ZIP10 was determined by WB analysis. **b.** Colony formation activity of Saos-2 cells with/without cisplatin (Cis) treatment. Data are means ± SEM; *P<0.05, **P<0.01.

**Fig. S5** ZIP10 overexpression promotes cell proliferation and chemoresistance in 143B cells. **a.** Left, the CCK-8 assay was performed to determine the proliferation of negative control (NC) and stable ZIP10-overexpressing (oeZIP10) 143B cells; right, the efficiency of oeZIP10 was determined by WB analysis. **b.** Colony formation activity of 143B cells with/without cisplatin (Cis) treatment. **c.** Animal experiments were performed to determine the proliferation and chemoresistance of negative control (NC) and stable ZIP10-overexpressing (oeZIP10) 143B cells. Scale bar = 1 cm. N = 6 mice per group. Data are means ± SEM; *P<0.05, **P<0.01.

**Fig. S6** Chemoresistance evaluation and gene expression of the cisplatin-resistant variant 143BR. **a.** A CCK-8 assay was performed to determine the proliferation of 143B and 143BR cells. **b.** Cell morphology of 143B and 143BR cells under a microscope. **c.** Flow cytometry analysis of apoptosis of 143B and 143BR cells with/without cisplatin (cis), adriamycin (ADR) and methotrexate (MTX) treatment. **d.** qRT-PCR and WB results confirmed that the ZIP10-ITGA10-p-AKT axis was activated in 143BR cells. Data are means ± SEM; *P<0.05, **P<0.01.

**Fig. S7** ZIP10 knockdown inhibits cell proliferation and chemoresistance in 143BR cells. **a**. Left, the CCK-8 assay was performed to determine the proliferation of negative control (NC) and stable ZIP10-knockdown (shZIP10) 143BR cells; right, the efficiency of shRNA targeting ZIP10 was determined by WB analysis. **b.** Colony formation activity of 143B cells with/without cisplatin (Cis) treatment **c.** Animal experiments were performed to determine the proliferation and chemoresistance of negative control (NC) and stable ZIP10-knockdown (shZIP10) 143BR cells. Scale bar = 1 cm. N = 6 mice per group. Data are means ± SEM; *P<0.05, **P<0.01.

**Fig. S8** Gene array analysis of NC and shZIP10 Saos-2 cells. Upregulated genes associated with the PI3K/AKT signaling pathway are shown.

**Fig. S9** Quantification of signaling pathways based on WB. The expression levels of p-AKT, p-ERK and p-JNK in 143B and Saos-2 cells with/without ZIP10 knockdown were quantified. Data are means ± SEM; *P<0.05, **P<0.01.

**Fig. S10** Knockdown of ZIP10 inhibits PI3K/AKT-mediated cell proliferation and chemoresistance in Saos-2 cells. **a**. ZIP10 knockdown (shZIP10), ZIP10 overexpression (oeZIP10), AKT activation (SC79) and AKT inhibitor (GSK690693) were applied to Saos-2 cells treated with DMSO (Con) or cisplatin (Cis) for 72 h. Flow cytometry was conducted to determine apoptosis. **b.** The whole-cell extract of Saos-2 cells treated with cisplatin for 72 h was subjected to WB analysis. **c.** EdU incorporation assay determination of Saos-2 cell proliferation. Scale bar, 20 μm. Data are means ± SEM; *P<0.05, **P<0.01.

**Fig. S11** qRT-PCR analysis of integrin expression in Saos-2 cells with/without ZIP10 knockdown. Expression of ITGA2, ITGA3 and ITGB8 was detected. Data are means ± SEM; *P<0.05, **P<0.01.

**Fig. S12** Flow cytometry analysis of cisplatin-induced apoptosis in 143B cells with/without ZIP overexpression or ITGA10 knockdown.

**Fig. S13** The effect of Zn on the proliferation and chemoresistance of 143B cells and Saos-2 cells. **a, b.** CCK8 was used to determine the proliferation of 143B cells and Saos-2 cells treated with ZnSO_4_ (**a;** 0, 5, 10, 15, 30, 50, 100, 200 µM) and TPEN (**b;** 0, 1, 3, 5, 10, 20 µM). **c.** Flow cytometry was conducted to determine cisplatin-induced apoptosis of 143B and Saos-2 cells treated with 15 µM ZnSO_4_ and 3 µM TPEN. Data are means ± SEM; *P<0.05, **P<0.01.

**Fig. S14** IHC staining analysis of Ki67, ZIP10, ITGA10, p-AKT and cleaved caspase 3 in xenograft tissues without cisplatin treatment. Data are means ± SEM; *P<0.05, **P<0.01.

**Fig. S15** The ZIP10-ITGA10-p-AKT signaling is required for cisplatin resistance in 143BR. **a-c.** Photographed xenograft tumors (**a**), average tumor volume (**b**) and tumor weight (**c**) of 143BR-NC, 143BR-shZIP10 and 143BR-shZIP10 + SC79 xenografts treated with vehicle (Con) or cisplatin (Cis). Scale bar, 1 cm. N = 6 mice per group. **d.** WB analysis was performed to detect target proteins, as indicated in xenograft tissues. **e.** IHC staining analysis of ZIP10, ITGA10, p-AKT, Ki67 and C-caspase-3 in xenograft tissues. Scale bar, 20 μm. Data are means ± SEM; *P<0.05, **P<0.01.
