# Supplementary figures and images for: ZIP10 drives osteosarcoma proliferation and chemoresistance through ITGA10-mediated activation of the PI3K/AKT pathway
Source: J Exp Clin Cancer Res. 2021 Oct 27;40:340. doi: 10.1186/s13046-021-02146-8 (PMC8549349; doi:10.1186/s13046-021-02146-8)

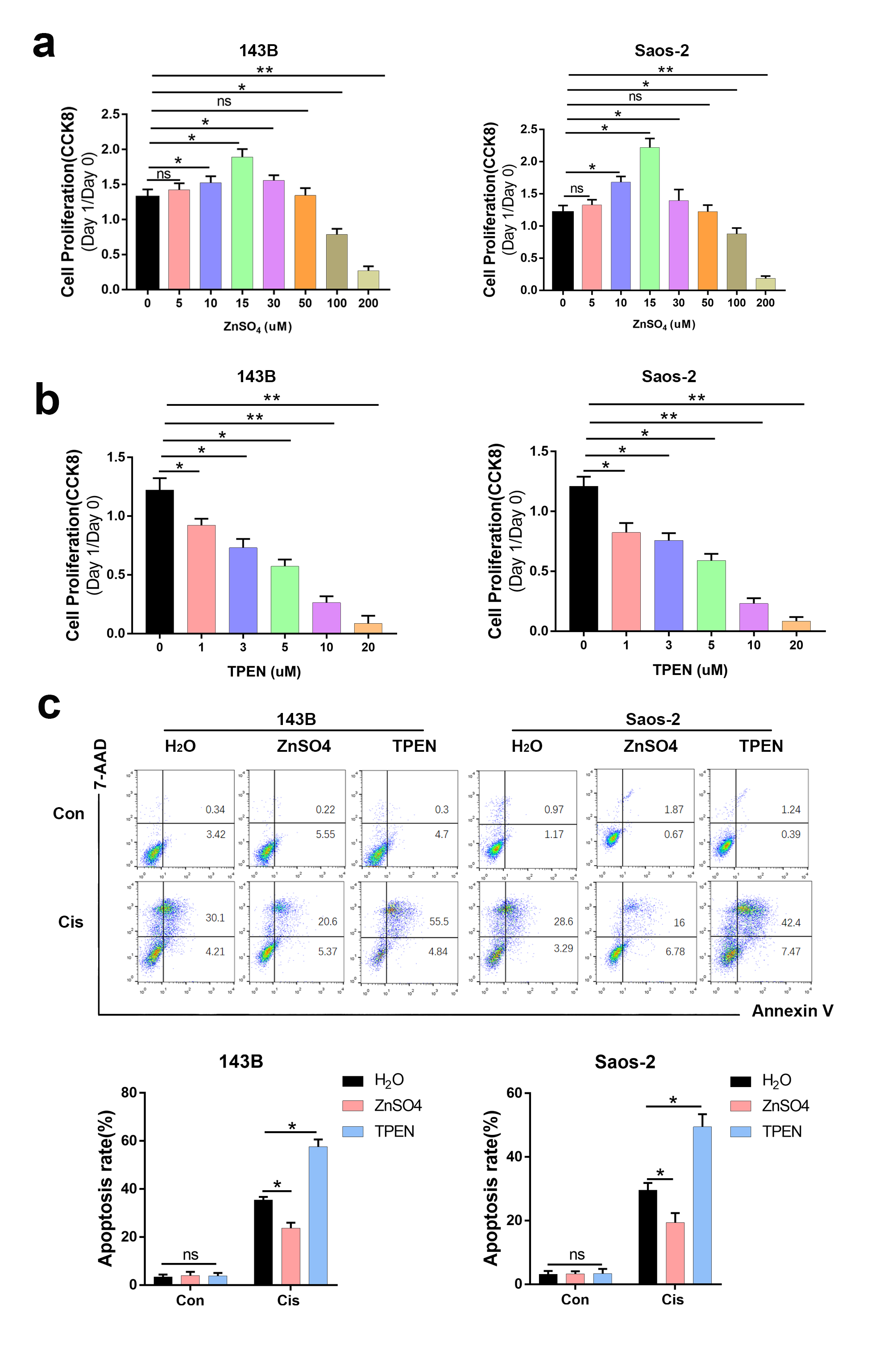

Supplement: Supplementary file 1 — Additional file 1: Fig. S1. Cisplatin induced OS cells apoptosis in a dose- and time-dependent manner. Fig. S2. qRT-PCR and WB analysis of ZIP10 expression in OS cells during cisplatin treatment. Fig. S3. Quantification of ZIP10 expression based on WB. Fig. S4. ZIP10 knockdown inhibits cell proliferation and chemoresistance in Saos-2 cells. Fig. S5. ZIP10 overexpression promotes cell proliferation and chemoresistance in 143B cells. Fig. S6. Chemoresistance evaluation and gene expression of the cisplatin-resistant variant 143BR. Fig. S7. ZIP10 knockdown inhibits cell proliferation and chemoresistance in 143BR cells. Fig. S8. Gene array analysis of NC and shZIP10 Saos-2 cells. Fig. S9. Quantification of signaling pathways based on WB. Fig. S10. Knockdown of ZIP10 inhibits PI3K/AKT-mediated cell proliferation and chemoresistance in Saos-2 cells. Fig. S11. qRT-PCR analysis of integrin expression in Saos-2 cells with/without ZIP10 knockdown. Fig. S12. Flow cytometry analysis of cisplatin-induced apoptosis in 143B cells with/without ZIP overexpression or ITGA10 knockdown. Fig. S13. The effect of Zn on the proliferation and chemoresistance of 143B cells and Saos-2 cells. Fig. S14. IHC staining analysis of Ki67, ZIP10, ITGA10, p-AKT and cleaved caspase 3 in xenograft tissues without cisplatin treatment. Fig. S15. The ZIP10-ITGA10-p-AKT signaling is required for cisplatin resistance in 143BR. [file 13046_2021_2146_MOESM1_ESM.zip › S13.tif]

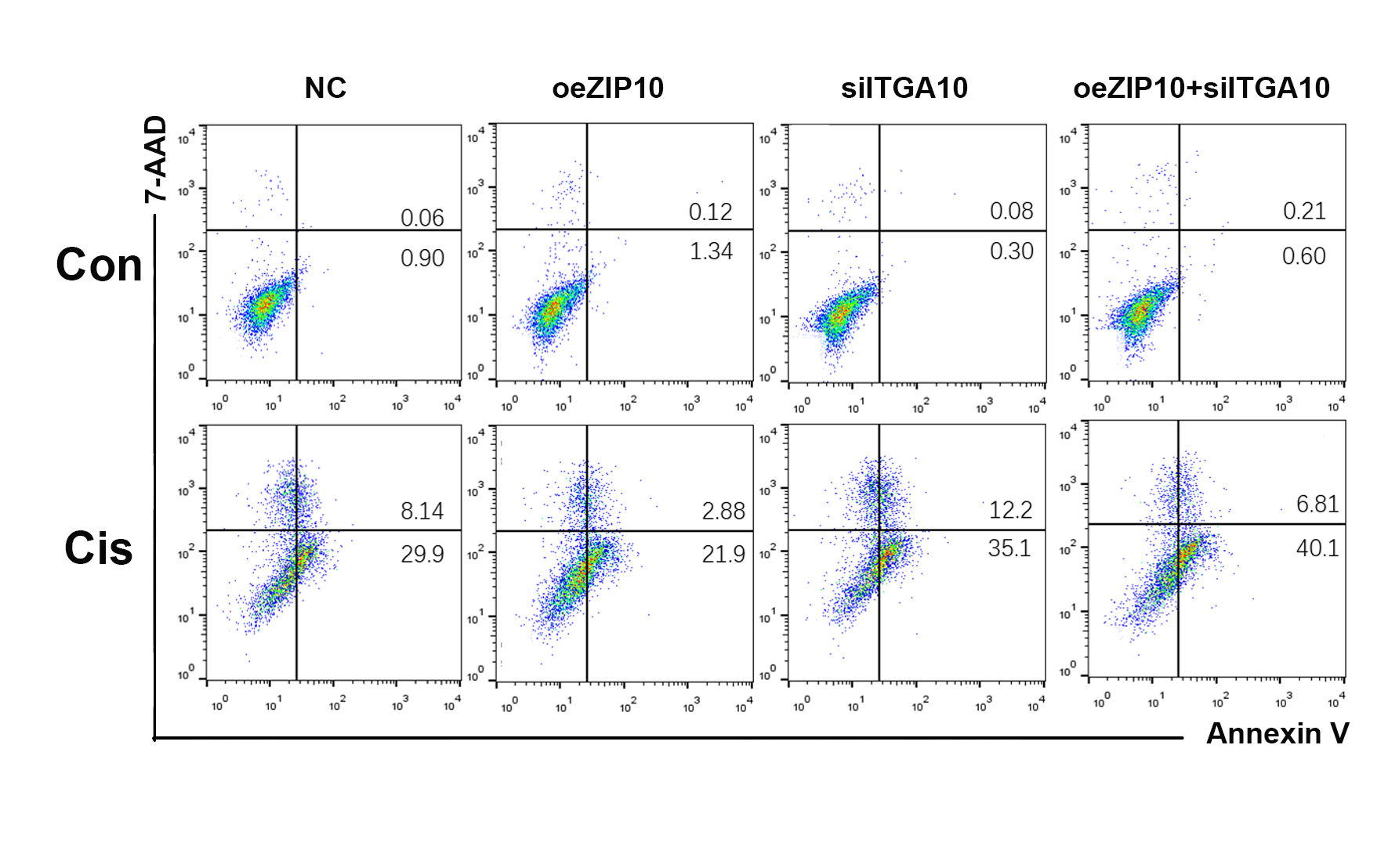

Supplement: Supplementary file 1 — Additional file 1: Fig. S1. Cisplatin induced OS cells apoptosis in a dose- and time-dependent manner. Fig. S2. qRT-PCR and WB analysis of ZIP10 expression in OS cells during cisplatin treatment. Fig. S3. Quantification of ZIP10 expression based on WB. Fig. S4. ZIP10 knockdown inhibits cell proliferation and chemoresistance in Saos-2 cells. Fig. S5. ZIP10 overexpression promotes cell proliferation and chemoresistance in 143B cells. Fig. S6. Chemoresistance evaluation and gene expression of the cisplatin-resistant variant 143BR. Fig. S7. ZIP10 knockdown inhibits cell proliferation and chemoresistance in 143BR cells. Fig. S8. Gene array analysis of NC and shZIP10 Saos-2 cells. Fig. S9. Quantification of signaling pathways based on WB. Fig. S10. Knockdown of ZIP10 inhibits PI3K/AKT-mediated cell proliferation and chemoresistance in Saos-2 cells. Fig. S11. qRT-PCR analysis of integrin expression in Saos-2 cells with/without ZIP10 knockdown. Fig. S12. Flow cytometry analysis of cisplatin-induced apoptosis in 143B cells with/without ZIP overexpression or ITGA10 knockdown. Fig. S13. The effect of Zn on the proliferation and chemoresistance of 143B cells and Saos-2 cells. Fig. S14. IHC staining analysis of Ki67, ZIP10, ITGA10, p-AKT and cleaved caspase 3 in xenograft tissues without cisplatin treatment. Fig. S15. The ZIP10-ITGA10-p-AKT signaling is required for cisplatin resistance in 143BR. [file 13046_2021_2146_MOESM1_ESM.zip › S12.tif]

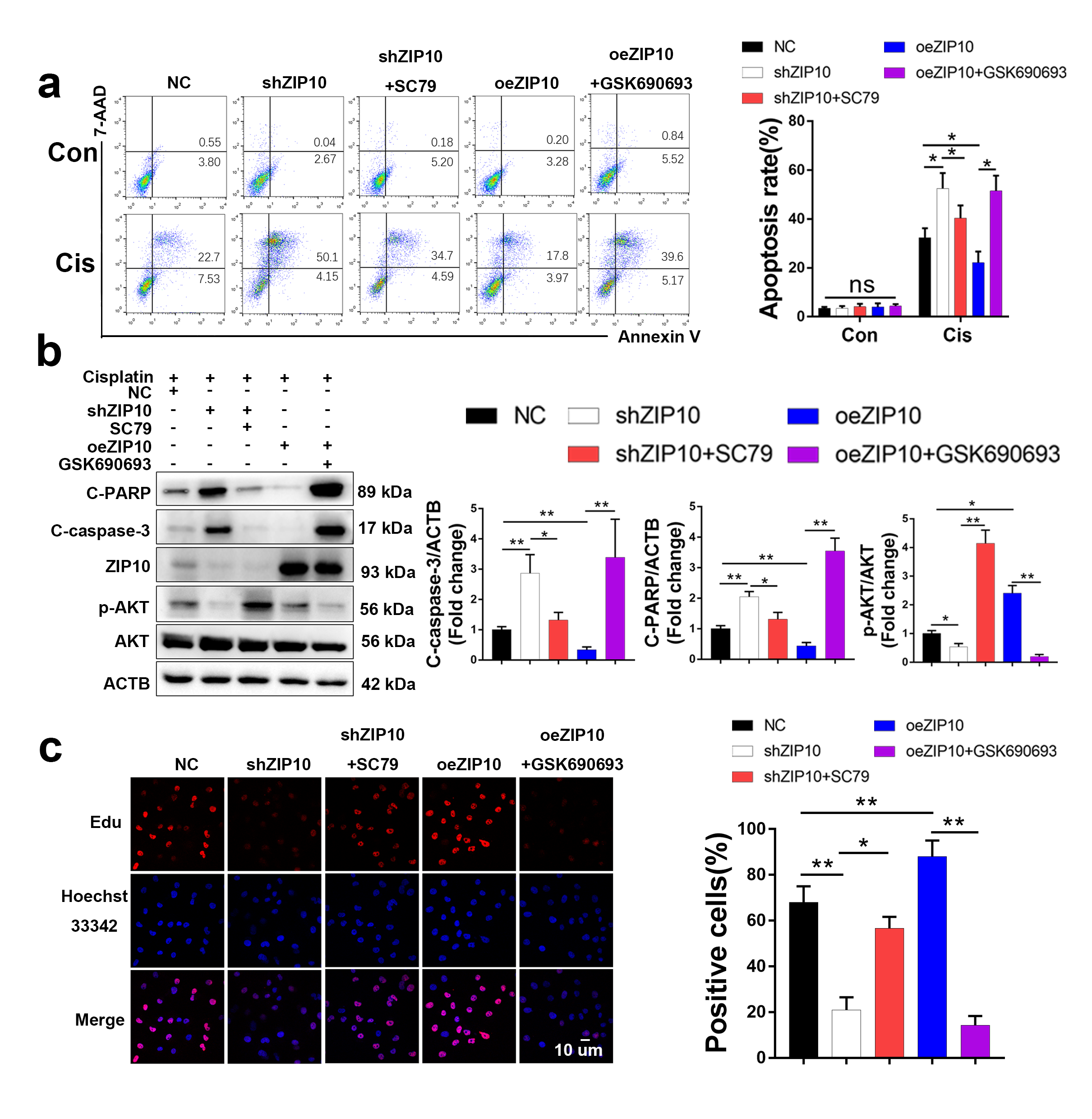

Supplement: Supplementary file 1 — Additional file 1: Fig. S1. Cisplatin induced OS cells apoptosis in a dose- and time-dependent manner. Fig. S2. qRT-PCR and WB analysis of ZIP10 expression in OS cells during cisplatin treatment. Fig. S3. Quantification of ZIP10 expression based on WB. Fig. S4. ZIP10 knockdown inhibits cell proliferation and chemoresistance in Saos-2 cells. Fig. S5. ZIP10 overexpression promotes cell proliferation and chemoresistance in 143B cells. Fig. S6. Chemoresistance evaluation and gene expression of the cisplatin-resistant variant 143BR. Fig. S7. ZIP10 knockdown inhibits cell proliferation and chemoresistance in 143BR cells. Fig. S8. Gene array analysis of NC and shZIP10 Saos-2 cells. Fig. S9. Quantification of signaling pathways based on WB. Fig. S10. Knockdown of ZIP10 inhibits PI3K/AKT-mediated cell proliferation and chemoresistance in Saos-2 cells. Fig. S11. qRT-PCR analysis of integrin expression in Saos-2 cells with/without ZIP10 knockdown. Fig. S12. Flow cytometry analysis of cisplatin-induced apoptosis in 143B cells with/without ZIP overexpression or ITGA10 knockdown. Fig. S13. The effect of Zn on the proliferation and chemoresistance of 143B cells and Saos-2 cells. Fig. S14. IHC staining analysis of Ki67, ZIP10, ITGA10, p-AKT and cleaved caspase 3 in xenograft tissues without cisplatin treatment. Fig. S15. The ZIP10-ITGA10-p-AKT signaling is required for cisplatin resistance in 143BR. [file 13046_2021_2146_MOESM1_ESM.zip › S10.tif]

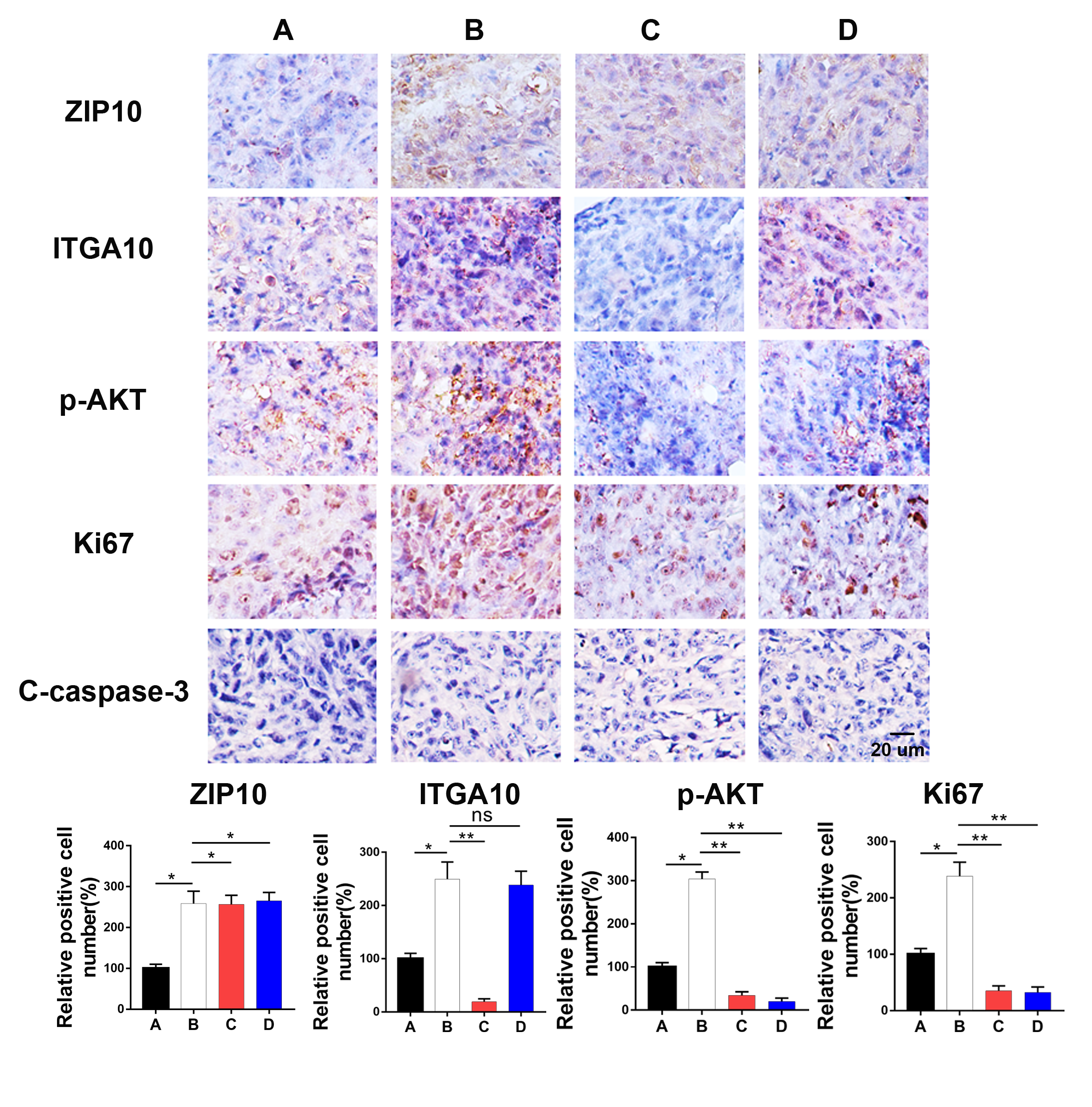

Supplement: Supplementary file 1 — Additional file 1: Fig. S1. Cisplatin induced OS cells apoptosis in a dose- and time-dependent manner. Fig. S2. qRT-PCR and WB analysis of ZIP10 expression in OS cells during cisplatin treatment. Fig. S3. Quantification of ZIP10 expression based on WB. Fig. S4. ZIP10 knockdown inhibits cell proliferation and chemoresistance in Saos-2 cells. Fig. S5. ZIP10 overexpression promotes cell proliferation and chemoresistance in 143B cells. Fig. S6. Chemoresistance evaluation and gene expression of the cisplatin-resistant variant 143BR. Fig. S7. ZIP10 knockdown inhibits cell proliferation and chemoresistance in 143BR cells. Fig. S8. Gene array analysis of NC and shZIP10 Saos-2 cells. Fig. S9. Quantification of signaling pathways based on WB. Fig. S10. Knockdown of ZIP10 inhibits PI3K/AKT-mediated cell proliferation and chemoresistance in Saos-2 cells. Fig. S11. qRT-PCR analysis of integrin expression in Saos-2 cells with/without ZIP10 knockdown. Fig. S12. Flow cytometry analysis of cisplatin-induced apoptosis in 143B cells with/without ZIP overexpression or ITGA10 knockdown. Fig. S13. The effect of Zn on the proliferation and chemoresistance of 143B cells and Saos-2 cells. Fig. S14. IHC staining analysis of Ki67, ZIP10, ITGA10, p-AKT and cleaved caspase 3 in xenograft tissues without cisplatin treatment. Fig. S15. The ZIP10-ITGA10-p-AKT signaling is required for cisplatin resistance in 143BR. [file 13046_2021_2146_MOESM1_ESM.zip › S14.tif]

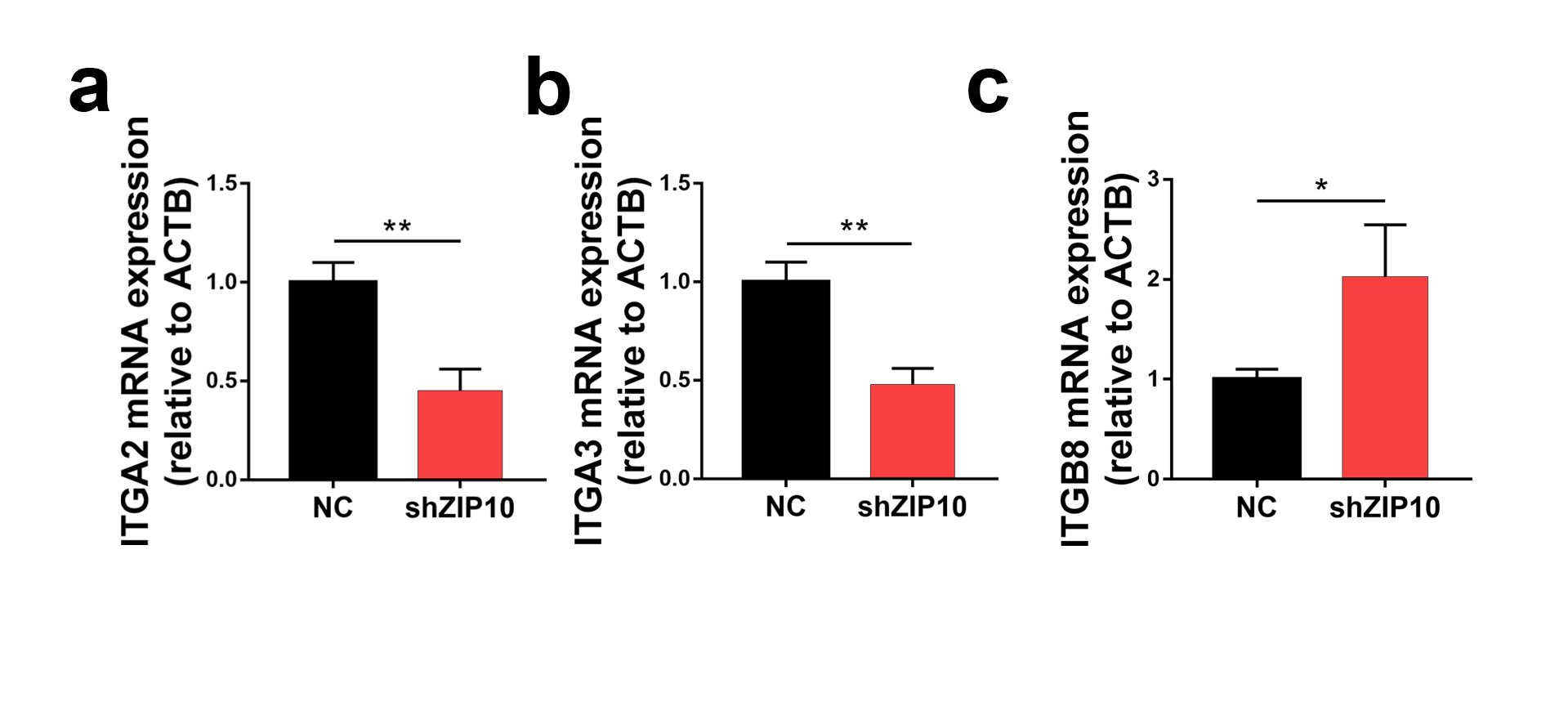

Supplement: Supplementary file 1 — Additional file 1: Fig. S1. Cisplatin induced OS cells apoptosis in a dose- and time-dependent manner. Fig. S2. qRT-PCR and WB analysis of ZIP10 expression in OS cells during cisplatin treatment. Fig. S3. Quantification of ZIP10 expression based on WB. Fig. S4. ZIP10 knockdown inhibits cell proliferation and chemoresistance in Saos-2 cells. Fig. S5. ZIP10 overexpression promotes cell proliferation and chemoresistance in 143B cells. Fig. S6. Chemoresistance evaluation and gene expression of the cisplatin-resistant variant 143BR. Fig. S7. ZIP10 knockdown inhibits cell proliferation and chemoresistance in 143BR cells. Fig. S8. Gene array analysis of NC and shZIP10 Saos-2 cells. Fig. S9. Quantification of signaling pathways based on WB. Fig. S10. Knockdown of ZIP10 inhibits PI3K/AKT-mediated cell proliferation and chemoresistance in Saos-2 cells. Fig. S11. qRT-PCR analysis of integrin expression in Saos-2 cells with/without ZIP10 knockdown. Fig. S12. Flow cytometry analysis of cisplatin-induced apoptosis in 143B cells with/without ZIP overexpression or ITGA10 knockdown. Fig. S13. The effect of Zn on the proliferation and chemoresistance of 143B cells and Saos-2 cells. Fig. S14. IHC staining analysis of Ki67, ZIP10, ITGA10, p-AKT and cleaved caspase 3 in xenograft tissues without cisplatin treatment. Fig. S15. The ZIP10-ITGA10-p-AKT signaling is required for cisplatin resistance in 143BR. [file 13046_2021_2146_MOESM1_ESM.zip › S11.tif]

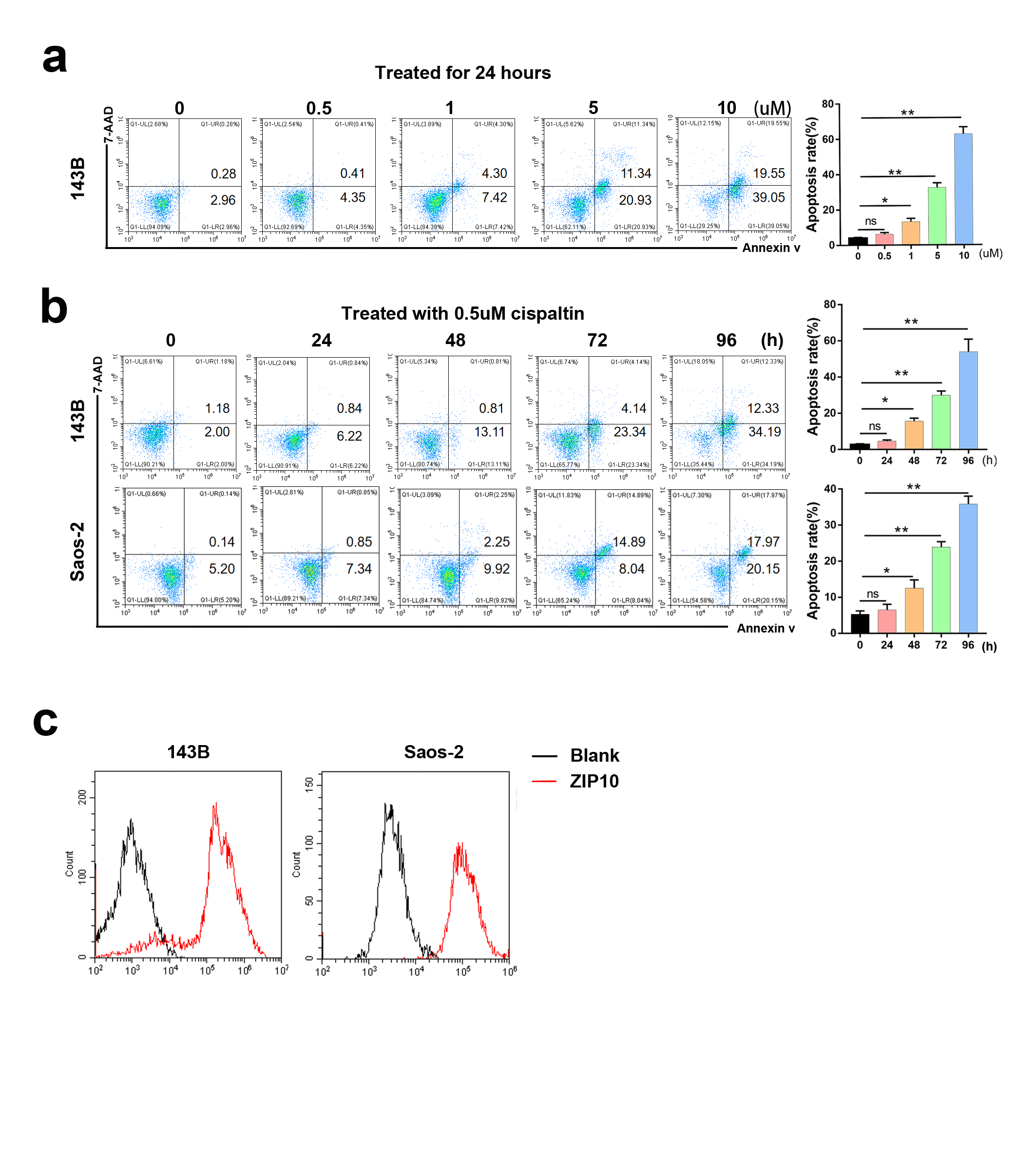

Supplement: Supplementary file 1 — Additional file 1: Fig. S1. Cisplatin induced OS cells apoptosis in a dose- and time-dependent manner. Fig. S2. qRT-PCR and WB analysis of ZIP10 expression in OS cells during cisplatin treatment. Fig. S3. Quantification of ZIP10 expression based on WB. Fig. S4. ZIP10 knockdown inhibits cell proliferation and chemoresistance in Saos-2 cells. Fig. S5. ZIP10 overexpression promotes cell proliferation and chemoresistance in 143B cells. Fig. S6. Chemoresistance evaluation and gene expression of the cisplatin-resistant variant 143BR. Fig. S7. ZIP10 knockdown inhibits cell proliferation and chemoresistance in 143BR cells. Fig. S8. Gene array analysis of NC and shZIP10 Saos-2 cells. Fig. S9. Quantification of signaling pathways based on WB. Fig. S10. Knockdown of ZIP10 inhibits PI3K/AKT-mediated cell proliferation and chemoresistance in Saos-2 cells. Fig. S11. qRT-PCR analysis of integrin expression in Saos-2 cells with/without ZIP10 knockdown. Fig. S12. Flow cytometry analysis of cisplatin-induced apoptosis in 143B cells with/without ZIP overexpression or ITGA10 knockdown. Fig. S13. The effect of Zn on the proliferation and chemoresistance of 143B cells and Saos-2 cells. Fig. S14. IHC staining analysis of Ki67, ZIP10, ITGA10, p-AKT and cleaved caspase 3 in xenograft tissues without cisplatin treatment. Fig. S15. The ZIP10-ITGA10-p-AKT signaling is required for cisplatin resistance in 143BR. [file 13046_2021_2146_MOESM1_ESM.zip › S1.tif]

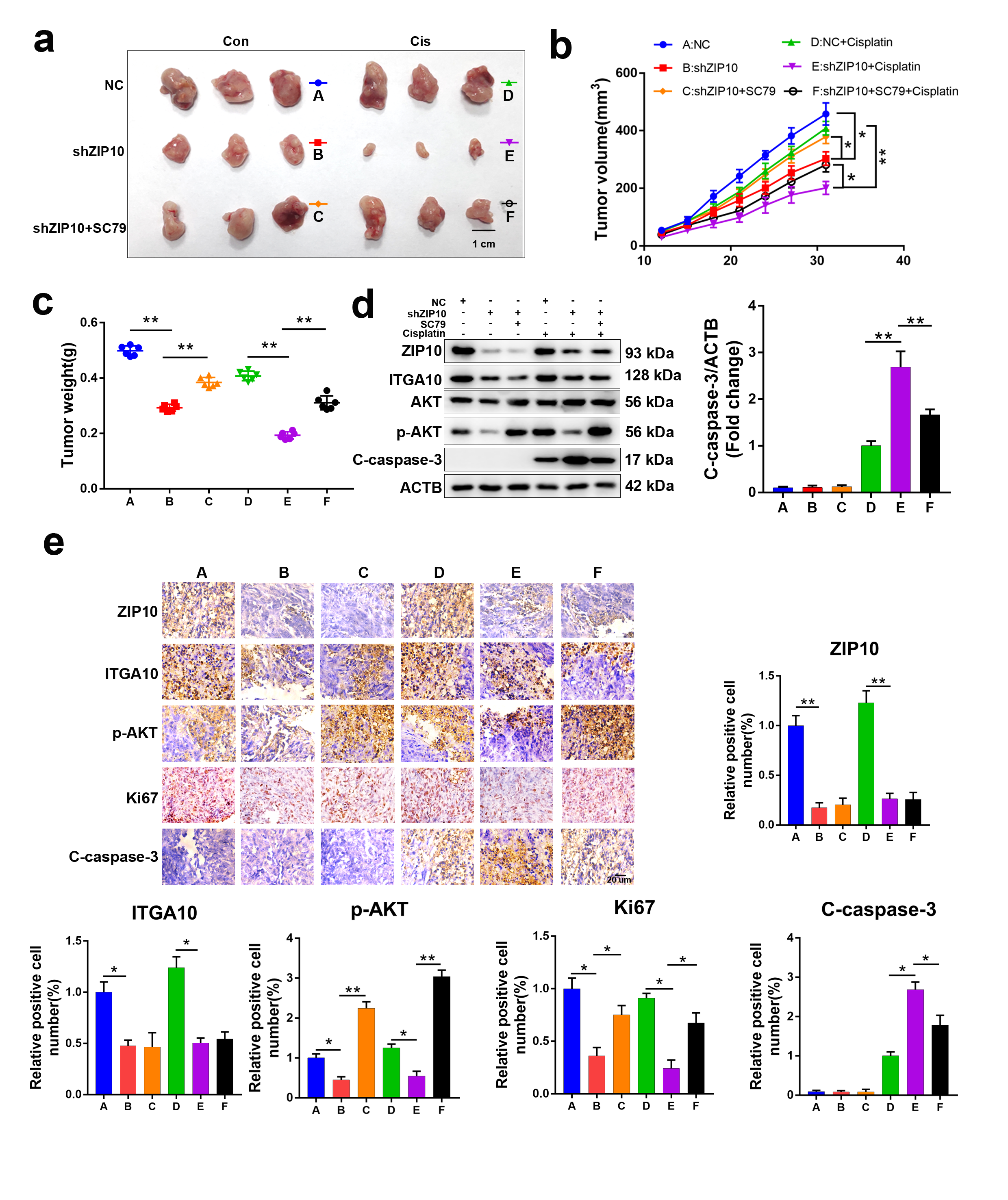

Supplement: Supplementary file 1 — Additional file 1: Fig. S1. Cisplatin induced OS cells apoptosis in a dose- and time-dependent manner. Fig. S2. qRT-PCR and WB analysis of ZIP10 expression in OS cells during cisplatin treatment. Fig. S3. Quantification of ZIP10 expression based on WB. Fig. S4. ZIP10 knockdown inhibits cell proliferation and chemoresistance in Saos-2 cells. Fig. S5. ZIP10 overexpression promotes cell proliferation and chemoresistance in 143B cells. Fig. S6. Chemoresistance evaluation and gene expression of the cisplatin-resistant variant 143BR. Fig. S7. ZIP10 knockdown inhibits cell proliferation and chemoresistance in 143BR cells. Fig. S8. Gene array analysis of NC and shZIP10 Saos-2 cells. Fig. S9. Quantification of signaling pathways based on WB. Fig. S10. Knockdown of ZIP10 inhibits PI3K/AKT-mediated cell proliferation and chemoresistance in Saos-2 cells. Fig. S11. qRT-PCR analysis of integrin expression in Saos-2 cells with/without ZIP10 knockdown. Fig. S12. Flow cytometry analysis of cisplatin-induced apoptosis in 143B cells with/without ZIP overexpression or ITGA10 knockdown. Fig. S13. The effect of Zn on the proliferation and chemoresistance of 143B cells and Saos-2 cells. Fig. S14. IHC staining analysis of Ki67, ZIP10, ITGA10, p-AKT and cleaved caspase 3 in xenograft tissues without cisplatin treatment. Fig. S15. The ZIP10-ITGA10-p-AKT signaling is required for cisplatin resistance in 143BR. [file 13046_2021_2146_MOESM1_ESM.zip › S15.tif]

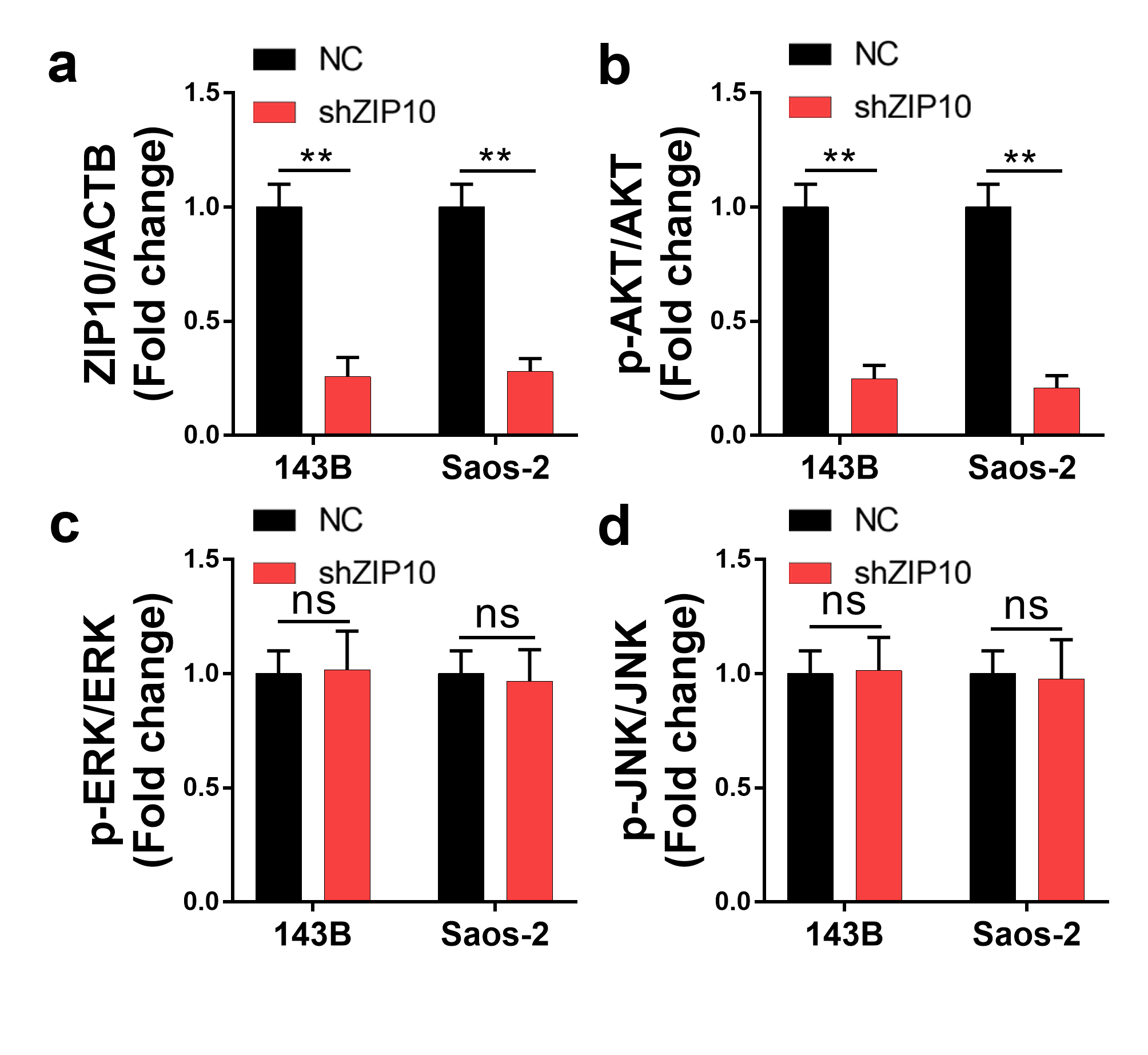

Supplement: Supplementary file 1 — Additional file 1: Fig. S1. Cisplatin induced OS cells apoptosis in a dose- and time-dependent manner. Fig. S2. qRT-PCR and WB analysis of ZIP10 expression in OS cells during cisplatin treatment. Fig. S3. Quantification of ZIP10 expression based on WB. Fig. S4. ZIP10 knockdown inhibits cell proliferation and chemoresistance in Saos-2 cells. Fig. S5. ZIP10 overexpression promotes cell proliferation and chemoresistance in 143B cells. Fig. S6. Chemoresistance evaluation and gene expression of the cisplatin-resistant variant 143BR. Fig. S7. ZIP10 knockdown inhibits cell proliferation and chemoresistance in 143BR cells. Fig. S8. Gene array analysis of NC and shZIP10 Saos-2 cells. Fig. S9. Quantification of signaling pathways based on WB. Fig. S10. Knockdown of ZIP10 inhibits PI3K/AKT-mediated cell proliferation and chemoresistance in Saos-2 cells. Fig. S11. qRT-PCR analysis of integrin expression in Saos-2 cells with/without ZIP10 knockdown. Fig. S12. Flow cytometry analysis of cisplatin-induced apoptosis in 143B cells with/without ZIP overexpression or ITGA10 knockdown. Fig. S13. The effect of Zn on the proliferation and chemoresistance of 143B cells and Saos-2 cells. Fig. S14. IHC staining analysis of Ki67, ZIP10, ITGA10, p-AKT and cleaved caspase 3 in xenograft tissues without cisplatin treatment. Fig. S15. The ZIP10-ITGA10-p-AKT signaling is required for cisplatin resistance in 143BR. [file 13046_2021_2146_MOESM1_ESM.zip › S9.tif]

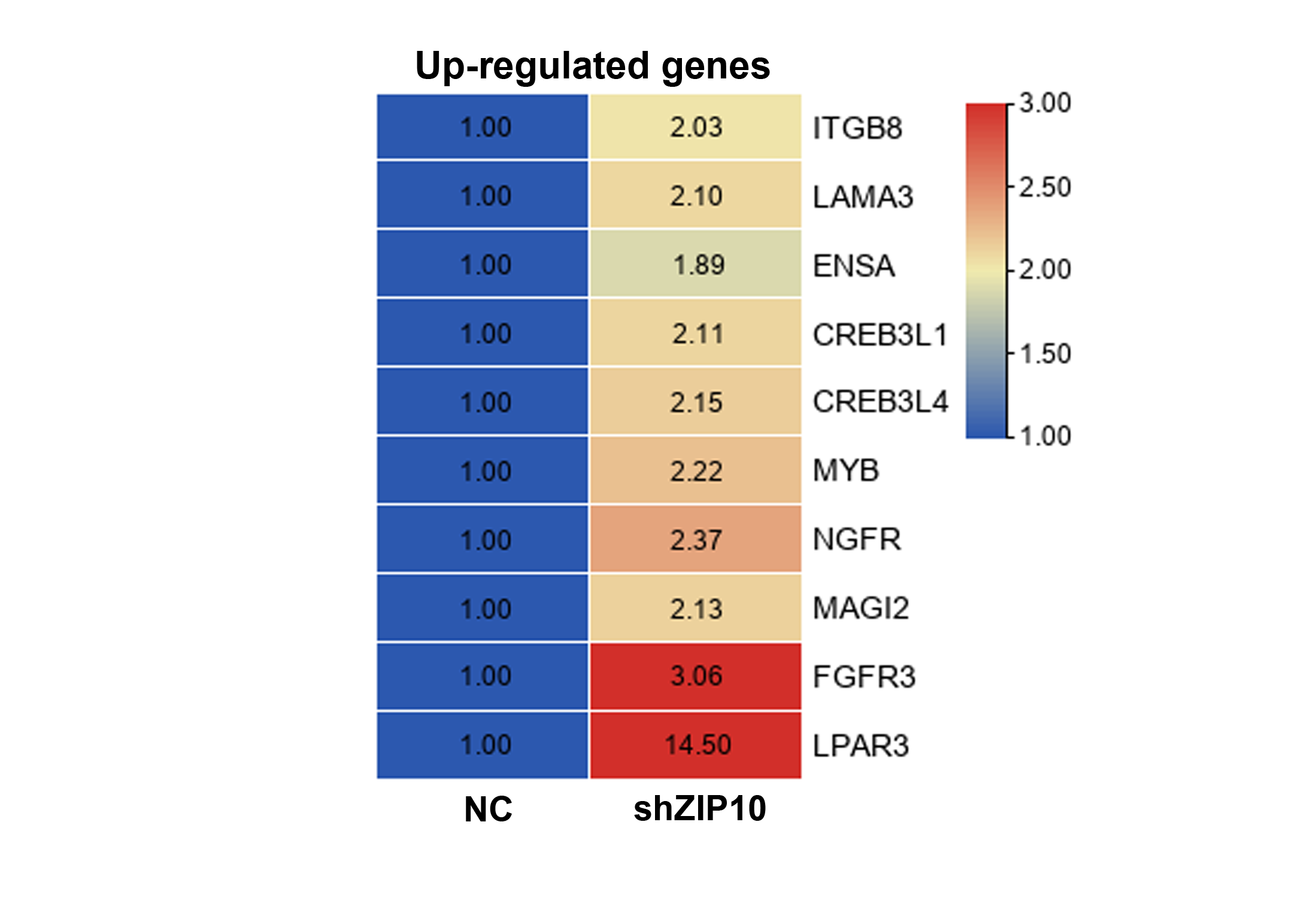

Supplement: Supplementary file 1 — Additional file 1: Fig. S1. Cisplatin induced OS cells apoptosis in a dose- and time-dependent manner. Fig. S2. qRT-PCR and WB analysis of ZIP10 expression in OS cells during cisplatin treatment. Fig. S3. Quantification of ZIP10 expression based on WB. Fig. S4. ZIP10 knockdown inhibits cell proliferation and chemoresistance in Saos-2 cells. Fig. S5. ZIP10 overexpression promotes cell proliferation and chemoresistance in 143B cells. Fig. S6. Chemoresistance evaluation and gene expression of the cisplatin-resistant variant 143BR. Fig. S7. ZIP10 knockdown inhibits cell proliferation and chemoresistance in 143BR cells. Fig. S8. Gene array analysis of NC and shZIP10 Saos-2 cells. Fig. S9. Quantification of signaling pathways based on WB. Fig. S10. Knockdown of ZIP10 inhibits PI3K/AKT-mediated cell proliferation and chemoresistance in Saos-2 cells. Fig. S11. qRT-PCR analysis of integrin expression in Saos-2 cells with/without ZIP10 knockdown. Fig. S12. Flow cytometry analysis of cisplatin-induced apoptosis in 143B cells with/without ZIP overexpression or ITGA10 knockdown. Fig. S13. The effect of Zn on the proliferation and chemoresistance of 143B cells and Saos-2 cells. Fig. S14. IHC staining analysis of Ki67, ZIP10, ITGA10, p-AKT and cleaved caspase 3 in xenograft tissues without cisplatin treatment. Fig. S15. The ZIP10-ITGA10-p-AKT signaling is required for cisplatin resistance in 143BR. [file 13046_2021_2146_MOESM1_ESM.zip › S8.tif]

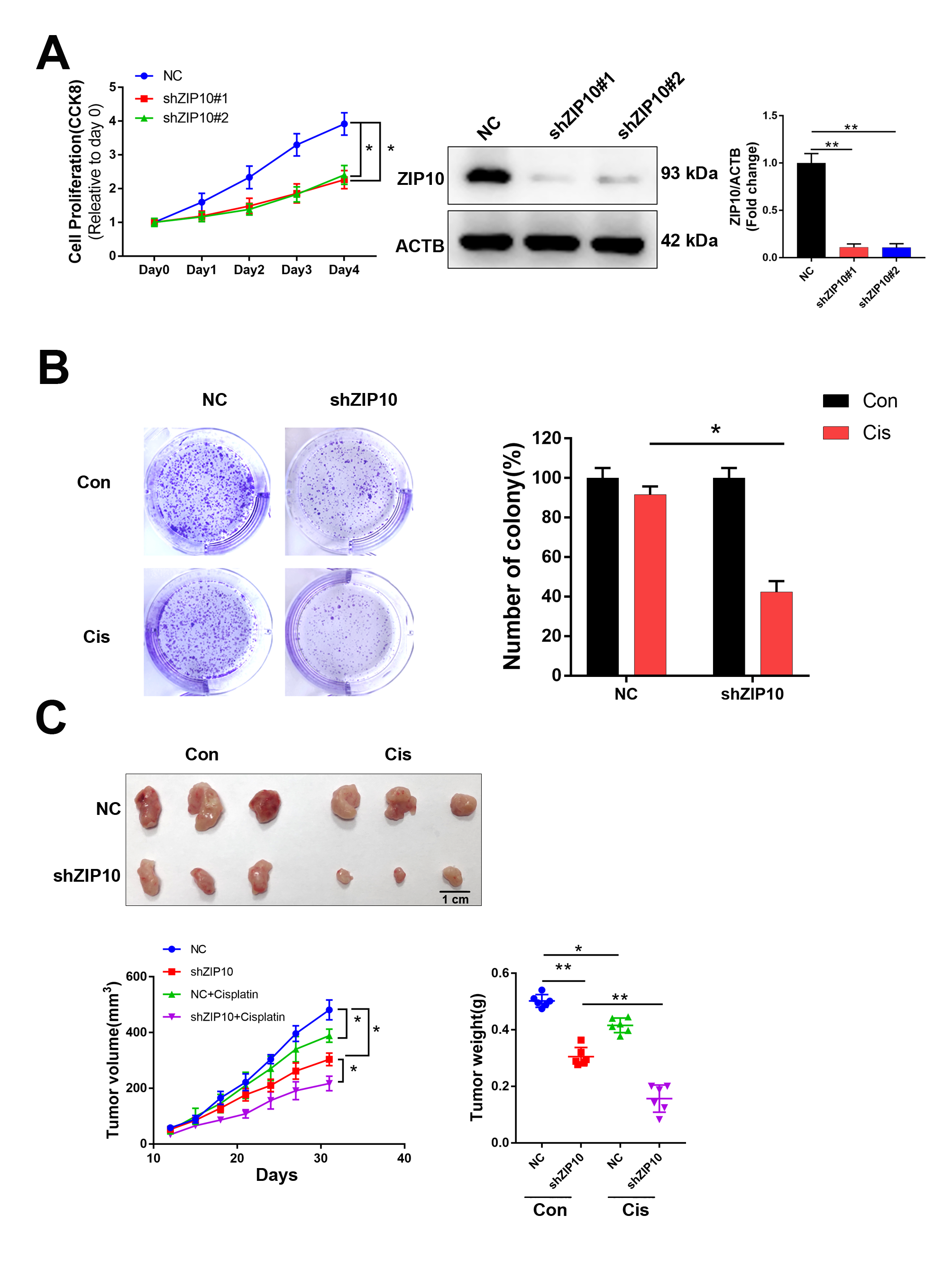

Supplement: Supplementary file 1 — Additional file 1: Fig. S1. Cisplatin induced OS cells apoptosis in a dose- and time-dependent manner. Fig. S2. qRT-PCR and WB analysis of ZIP10 expression in OS cells during cisplatin treatment. Fig. S3. Quantification of ZIP10 expression based on WB. Fig. S4. ZIP10 knockdown inhibits cell proliferation and chemoresistance in Saos-2 cells. Fig. S5. ZIP10 overexpression promotes cell proliferation and chemoresistance in 143B cells. Fig. S6. Chemoresistance evaluation and gene expression of the cisplatin-resistant variant 143BR. Fig. S7. ZIP10 knockdown inhibits cell proliferation and chemoresistance in 143BR cells. Fig. S8. Gene array analysis of NC and shZIP10 Saos-2 cells. Fig. S9. Quantification of signaling pathways based on WB. Fig. S10. Knockdown of ZIP10 inhibits PI3K/AKT-mediated cell proliferation and chemoresistance in Saos-2 cells. Fig. S11. qRT-PCR analysis of integrin expression in Saos-2 cells with/without ZIP10 knockdown. Fig. S12. Flow cytometry analysis of cisplatin-induced apoptosis in 143B cells with/without ZIP overexpression or ITGA10 knockdown. Fig. S13. The effect of Zn on the proliferation and chemoresistance of 143B cells and Saos-2 cells. Fig. S14. IHC staining analysis of Ki67, ZIP10, ITGA10, p-AKT and cleaved caspase 3 in xenograft tissues without cisplatin treatment. Fig. S15. The ZIP10-ITGA10-p-AKT signaling is required for cisplatin resistance in 143BR. [file 13046_2021_2146_MOESM1_ESM.zip › S7.tif]

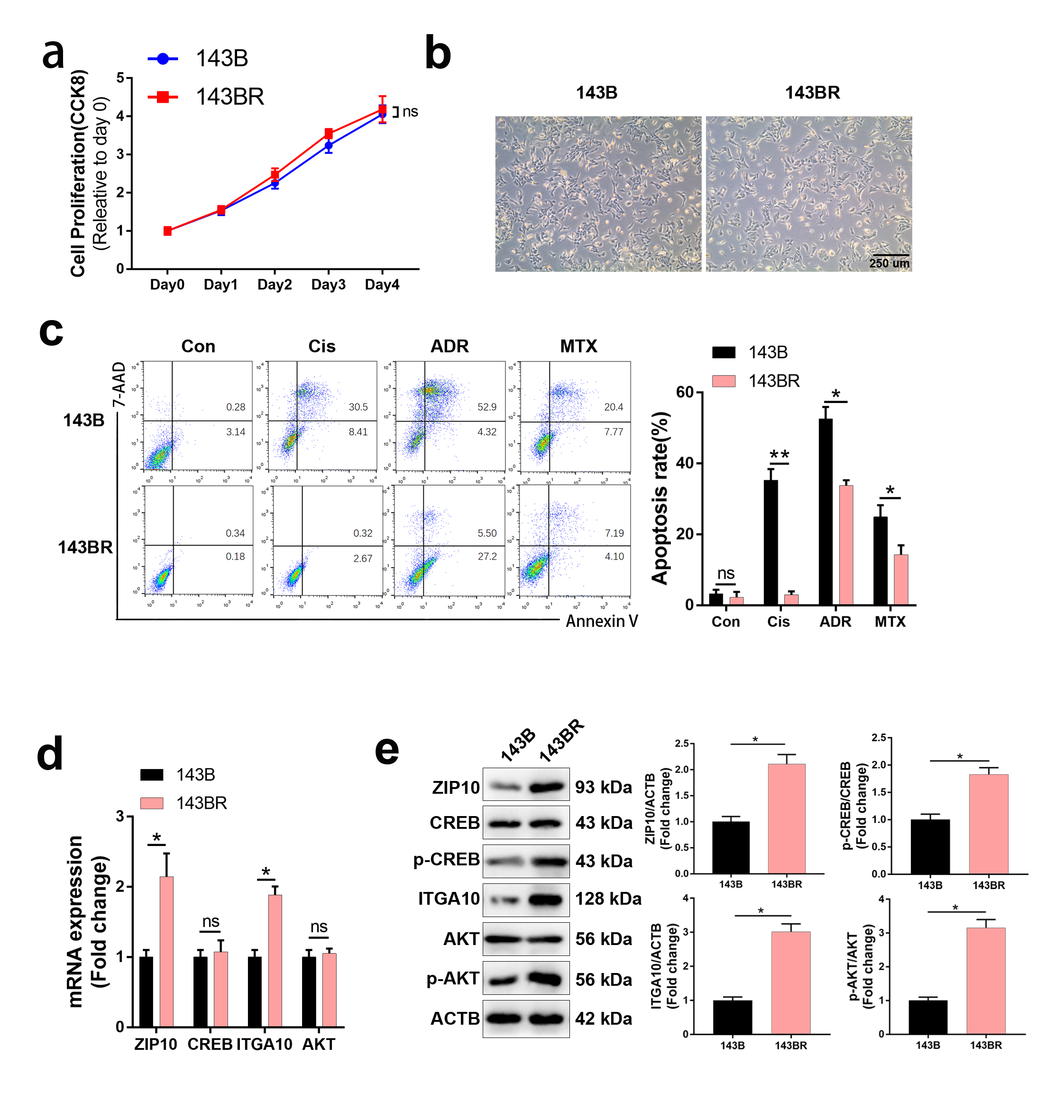

Supplement: Supplementary file 1 — Additional file 1: Fig. S1. Cisplatin induced OS cells apoptosis in a dose- and time-dependent manner. Fig. S2. qRT-PCR and WB analysis of ZIP10 expression in OS cells during cisplatin treatment. Fig. S3. Quantification of ZIP10 expression based on WB. Fig. S4. ZIP10 knockdown inhibits cell proliferation and chemoresistance in Saos-2 cells. Fig. S5. ZIP10 overexpression promotes cell proliferation and chemoresistance in 143B cells. Fig. S6. Chemoresistance evaluation and gene expression of the cisplatin-resistant variant 143BR. Fig. S7. ZIP10 knockdown inhibits cell proliferation and chemoresistance in 143BR cells. Fig. S8. Gene array analysis of NC and shZIP10 Saos-2 cells. Fig. S9. Quantification of signaling pathways based on WB. Fig. S10. Knockdown of ZIP10 inhibits PI3K/AKT-mediated cell proliferation and chemoresistance in Saos-2 cells. Fig. S11. qRT-PCR analysis of integrin expression in Saos-2 cells with/without ZIP10 knockdown. Fig. S12. Flow cytometry analysis of cisplatin-induced apoptosis in 143B cells with/without ZIP overexpression or ITGA10 knockdown. Fig. S13. The effect of Zn on the proliferation and chemoresistance of 143B cells and Saos-2 cells. Fig. S14. IHC staining analysis of Ki67, ZIP10, ITGA10, p-AKT and cleaved caspase 3 in xenograft tissues without cisplatin treatment. Fig. S15. The ZIP10-ITGA10-p-AKT signaling is required for cisplatin resistance in 143BR. [file 13046_2021_2146_MOESM1_ESM.zip › S6.tif]

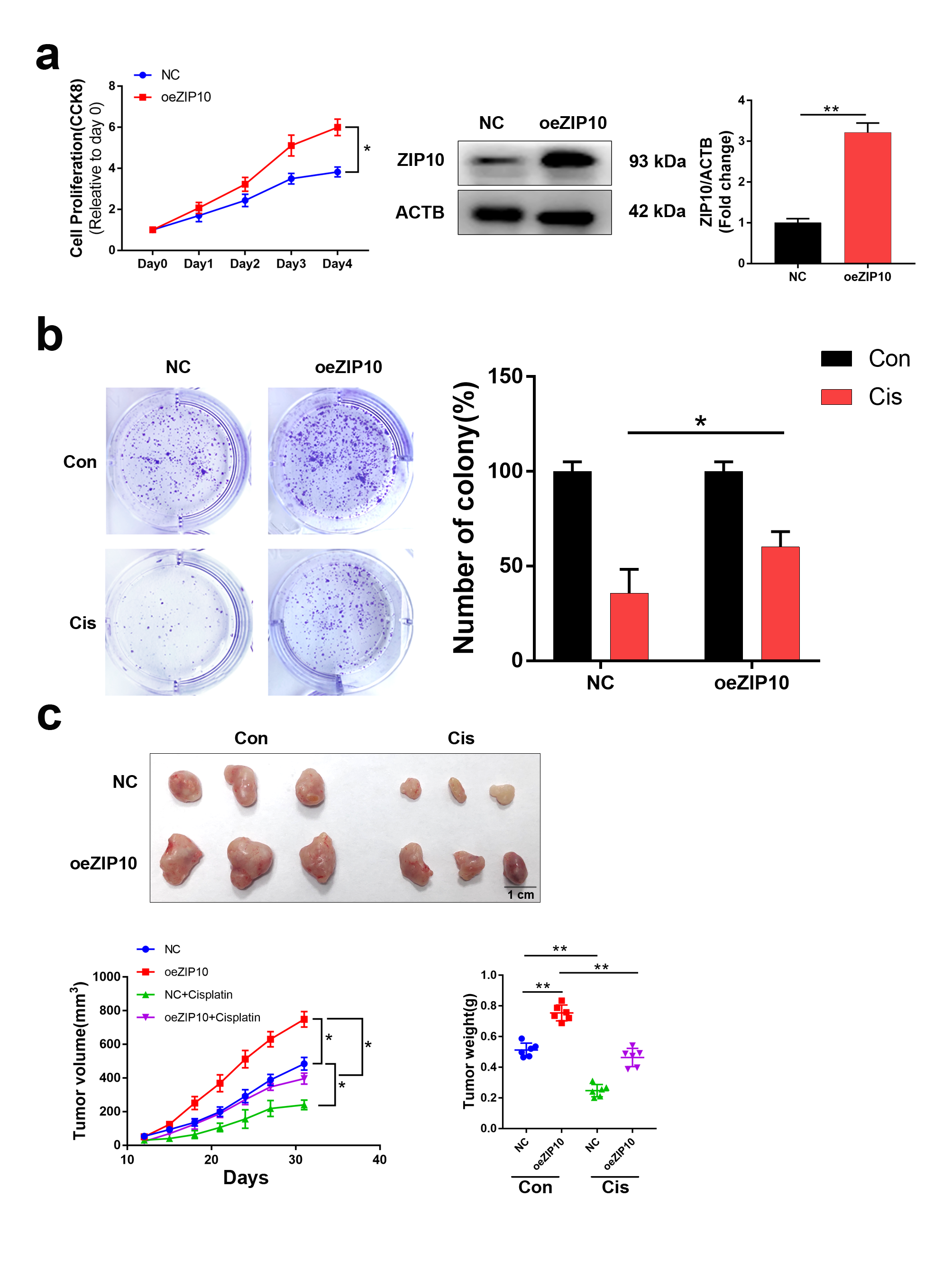

Supplement: Supplementary file 1 — Additional file 1: Fig. S1. Cisplatin induced OS cells apoptosis in a dose- and time-dependent manner. Fig. S2. qRT-PCR and WB analysis of ZIP10 expression in OS cells during cisplatin treatment. Fig. S3. Quantification of ZIP10 expression based on WB. Fig. S4. ZIP10 knockdown inhibits cell proliferation and chemoresistance in Saos-2 cells. Fig. S5. ZIP10 overexpression promotes cell proliferation and chemoresistance in 143B cells. Fig. S6. Chemoresistance evaluation and gene expression of the cisplatin-resistant variant 143BR. Fig. S7. ZIP10 knockdown inhibits cell proliferation and chemoresistance in 143BR cells. Fig. S8. Gene array analysis of NC and shZIP10 Saos-2 cells. Fig. S9. Quantification of signaling pathways based on WB. Fig. S10. Knockdown of ZIP10 inhibits PI3K/AKT-mediated cell proliferation and chemoresistance in Saos-2 cells. Fig. S11. qRT-PCR analysis of integrin expression in Saos-2 cells with/without ZIP10 knockdown. Fig. S12. Flow cytometry analysis of cisplatin-induced apoptosis in 143B cells with/without ZIP overexpression or ITGA10 knockdown. Fig. S13. The effect of Zn on the proliferation and chemoresistance of 143B cells and Saos-2 cells. Fig. S14. IHC staining analysis of Ki67, ZIP10, ITGA10, p-AKT and cleaved caspase 3 in xenograft tissues without cisplatin treatment. Fig. S15. The ZIP10-ITGA10-p-AKT signaling is required for cisplatin resistance in 143BR. [file 13046_2021_2146_MOESM1_ESM.zip › S5.tif]

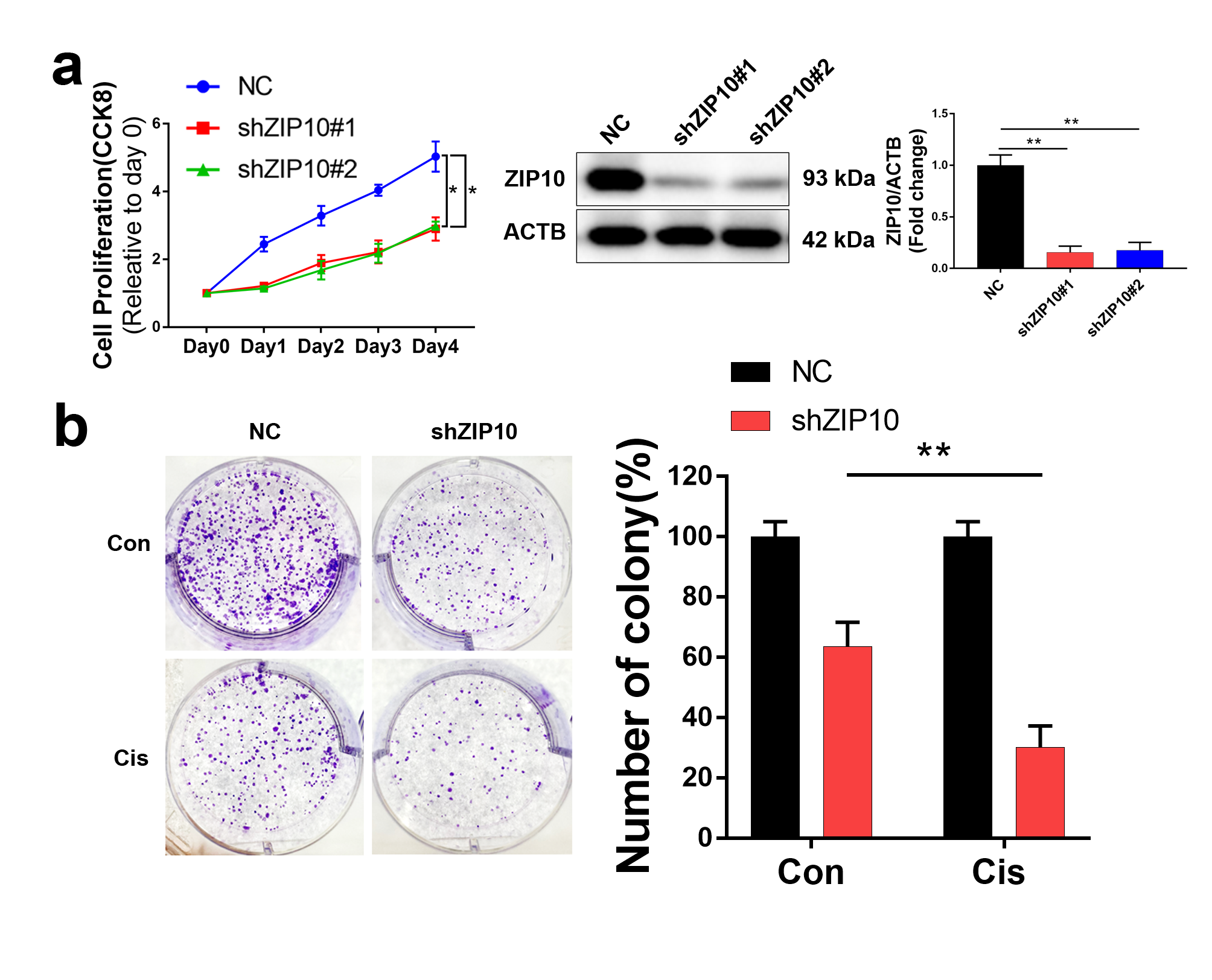

Supplement: Supplementary file 1 — Additional file 1: Fig. S1. Cisplatin induced OS cells apoptosis in a dose- and time-dependent manner. Fig. S2. qRT-PCR and WB analysis of ZIP10 expression in OS cells during cisplatin treatment. Fig. S3. Quantification of ZIP10 expression based on WB. Fig. S4. ZIP10 knockdown inhibits cell proliferation and chemoresistance in Saos-2 cells. Fig. S5. ZIP10 overexpression promotes cell proliferation and chemoresistance in 143B cells. Fig. S6. Chemoresistance evaluation and gene expression of the cisplatin-resistant variant 143BR. Fig. S7. ZIP10 knockdown inhibits cell proliferation and chemoresistance in 143BR cells. Fig. S8. Gene array analysis of NC and shZIP10 Saos-2 cells. Fig. S9. Quantification of signaling pathways based on WB. Fig. S10. Knockdown of ZIP10 inhibits PI3K/AKT-mediated cell proliferation and chemoresistance in Saos-2 cells. Fig. S11. qRT-PCR analysis of integrin expression in Saos-2 cells with/without ZIP10 knockdown. Fig. S12. Flow cytometry analysis of cisplatin-induced apoptosis in 143B cells with/without ZIP overexpression or ITGA10 knockdown. Fig. S13. The effect of Zn on the proliferation and chemoresistance of 143B cells and Saos-2 cells. Fig. S14. IHC staining analysis of Ki67, ZIP10, ITGA10, p-AKT and cleaved caspase 3 in xenograft tissues without cisplatin treatment. Fig. S15. The ZIP10-ITGA10-p-AKT signaling is required for cisplatin resistance in 143BR. [file 13046_2021_2146_MOESM1_ESM.zip › S4.tif]

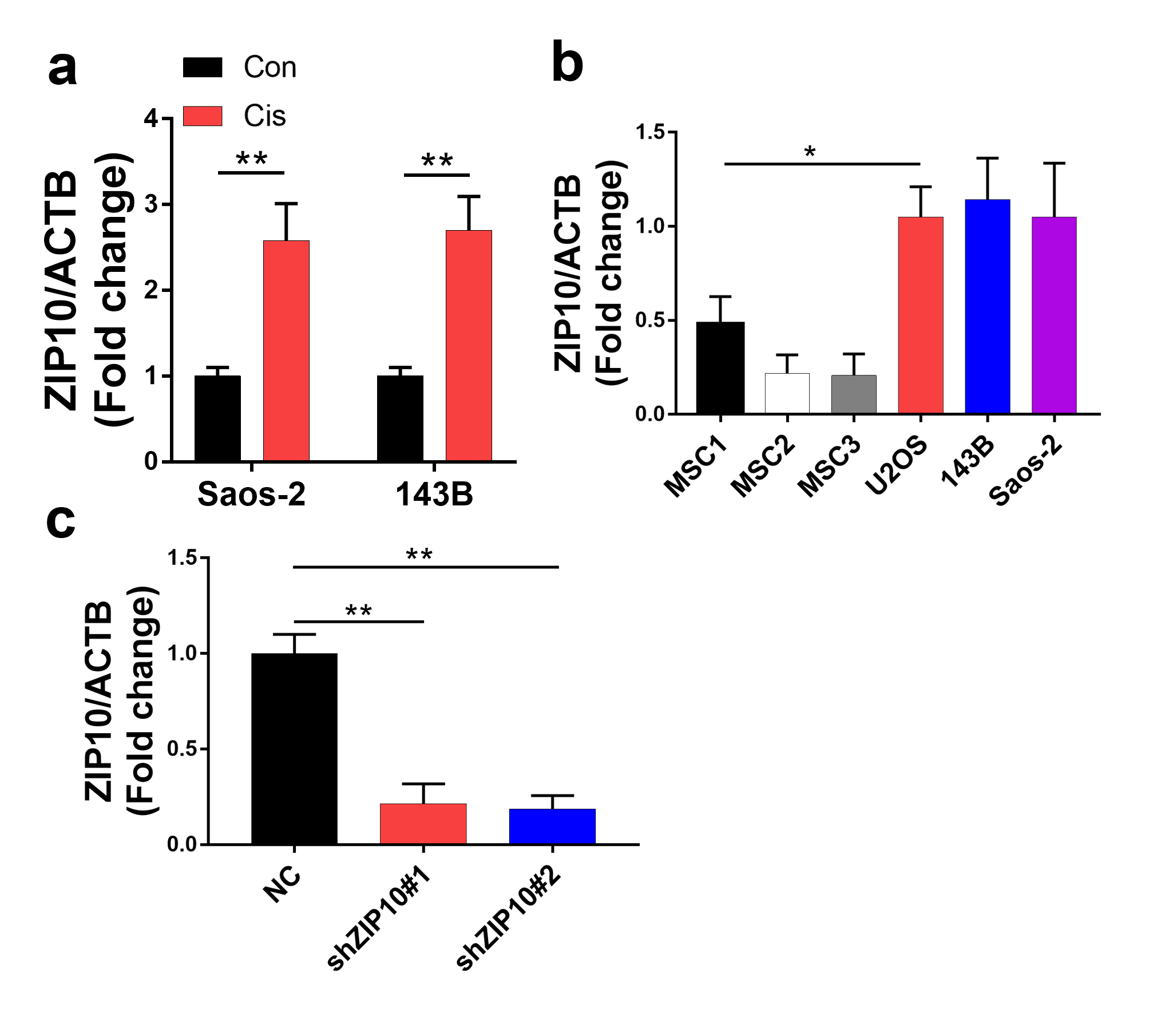

Supplement: Supplementary file 1 — Additional file 1: Fig. S1. Cisplatin induced OS cells apoptosis in a dose- and time-dependent manner. Fig. S2. qRT-PCR and WB analysis of ZIP10 expression in OS cells during cisplatin treatment. Fig. S3. Quantification of ZIP10 expression based on WB. Fig. S4. ZIP10 knockdown inhibits cell proliferation and chemoresistance in Saos-2 cells. Fig. S5. ZIP10 overexpression promotes cell proliferation and chemoresistance in 143B cells. Fig. S6. Chemoresistance evaluation and gene expression of the cisplatin-resistant variant 143BR. Fig. S7. ZIP10 knockdown inhibits cell proliferation and chemoresistance in 143BR cells. Fig. S8. Gene array analysis of NC and shZIP10 Saos-2 cells. Fig. S9. Quantification of signaling pathways based on WB. Fig. S10. Knockdown of ZIP10 inhibits PI3K/AKT-mediated cell proliferation and chemoresistance in Saos-2 cells. Fig. S11. qRT-PCR analysis of integrin expression in Saos-2 cells with/without ZIP10 knockdown. Fig. S12. Flow cytometry analysis of cisplatin-induced apoptosis in 143B cells with/without ZIP overexpression or ITGA10 knockdown. Fig. S13. The effect of Zn on the proliferation and chemoresistance of 143B cells and Saos-2 cells. Fig. S14. IHC staining analysis of Ki67, ZIP10, ITGA10, p-AKT and cleaved caspase 3 in xenograft tissues without cisplatin treatment. Fig. S15. The ZIP10-ITGA10-p-AKT signaling is required for cisplatin resistance in 143BR. [file 13046_2021_2146_MOESM1_ESM.zip › S3.tif]

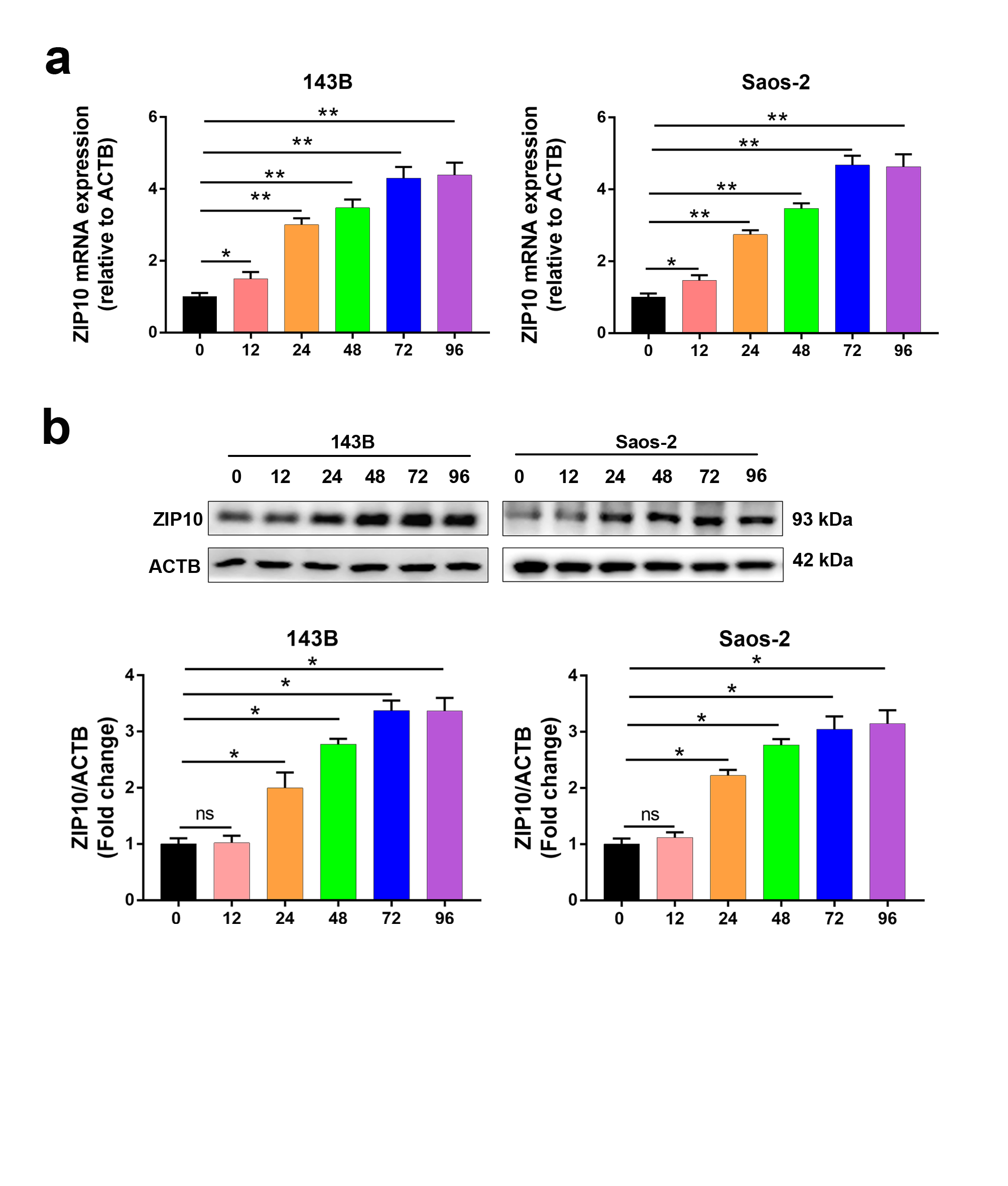

Supplement: Supplementary file 1 — Additional file 1: Fig. S1. Cisplatin induced OS cells apoptosis in a dose- and time-dependent manner. Fig. S2. qRT-PCR and WB analysis of ZIP10 expression in OS cells during cisplatin treatment. Fig. S3. Quantification of ZIP10 expression based on WB. Fig. S4. ZIP10 knockdown inhibits cell proliferation and chemoresistance in Saos-2 cells. Fig. S5. ZIP10 overexpression promotes cell proliferation and chemoresistance in 143B cells. Fig. S6. Chemoresistance evaluation and gene expression of the cisplatin-resistant variant 143BR. Fig. S7. ZIP10 knockdown inhibits cell proliferation and chemoresistance in 143BR cells. Fig. S8. Gene array analysis of NC and shZIP10 Saos-2 cells. Fig. S9. Quantification of signaling pathways based on WB. Fig. S10. Knockdown of ZIP10 inhibits PI3K/AKT-mediated cell proliferation and chemoresistance in Saos-2 cells. Fig. S11. qRT-PCR analysis of integrin expression in Saos-2 cells with/without ZIP10 knockdown. Fig. S12. Flow cytometry analysis of cisplatin-induced apoptosis in 143B cells with/without ZIP overexpression or ITGA10 knockdown. Fig. S13. The effect of Zn on the proliferation and chemoresistance of 143B cells and Saos-2 cells. Fig. S14. IHC staining analysis of Ki67, ZIP10, ITGA10, p-AKT and cleaved caspase 3 in xenograft tissues without cisplatin treatment. Fig. S15. The ZIP10-ITGA10-p-AKT signaling is required for cisplatin resistance in 143BR. [file 13046_2021_2146_MOESM1_ESM.zip › S2.tif]
